# Reference Intervals for Hemoglobin and Hematocrit Adjusted for Altitude, Sex, and Age: A Big Data-Based Study in the Colombian Population

**DOI:** 10.3390/medsci14010136

**Published:** 2026-03-14

**Authors:** Esteban Morales-Mendoza, María del Pilar Suarez-Ramos, Marcela Godoy-Corredor, Natalia Gomez-Lopera, Juan Felipe Combariza-Vallejo, Jossie Murcia, Mario A. Isaza-Ruget

**Affiliations:** 1 Fundación Universitaria Sanitas, Bogotá 111321, Colombia; juan.morales@unisanitas.edu.co (E.M.-M.); misaza@colsanitas.com (M.A.I.-R.); 2 Laboratorio Clínico y de Patología, Clínica Colsanitas, Bogotá 111321, Colombia; maridsuarez@colsanitas.com (M.d.P.S.-R.); ext.natalia.gomezl@keralty.com (N.G.-L.); 3 Clínica Universitaria Colombia, Clínica Colsanitas, Bogotá 111321, Colombia; jfcombariza@colsanitas.com

**Keywords:** hemoglobin, hematocrit, reference intervals, altitude, Colombia, big data, indirect method

## Abstract

**Background**: Hemoglobin (Hb) and hematocrit (Hct) reference intervals (RIs) are critical for diagnosing hematological disorders. However, existing reference values often do not account for demographic and environmental variability. Particularly in countries with altitude gradients, such as Colombia, the absence of locally adjusted intervals may lead to the misclassification of anemia and polycythemia. Therefore, this study aims to establish sex-, age-, and altitude-specific reference intervals for Hb and Hct within the Colombian adult population via an indirect, big-data-based methodology. **Methods**: This retrospective cross-sectional study used 3.1 million Hb and Hct test results nationwide between 2022 and 2024. After applying the exclusion criteria, Hb data from 667,857 individuals and Hct data from 662,024 individuals were included. The population was stratified by sex, age, and altitude into <1100 m above sea level (m.a.s.l.), 1100–2000 m.a.s.l., and 2000–3000 m.a.s.l. Reference intervals (RIs) were estimated via the refineR algorithm, and the results were compared across altitude categories and against World Health Organization (WHO) anemia and polycythemia thresholds. **Results**: Hb and Hct concentrations increased with altitude in all sexes and age groups. Compared with women, men presented higher mean values and narrower RIs, whereas older adults presented greater variability. Compared with WHO thresholds, a significant proportion of individuals living above 2000 m exceeded polycythemia cutoffs without clinical evidence of disease, suggesting the need for altitude-adjusted diagnostic criteria. **Conclusions**: This study provides the first large-scale, data-driven reference intervals for Hb and Hct in Colombia, adjusted for altitude, sex, and age. The implementation of locally derived RIs may improve diagnostic accuracy and prevent the over- or underdiagnosis of hematological disorders, with direct implications for clinical decision-making and public health policy.

## 1. Introduction

Hemoglobin (Hb) and hematocrit (Hct) are indicators of blood oxygen-carrying capacity, and their values are influenced by factors such as age, sex, ethnicity, and altitude [1,2]. At high elevations, the reduced ambient oxygen pressure stimulates red blood cell production, increasing Hb and Hct levels through a process known as compensatory erythrocytosis. The extent of this response depends on both altitude and duration of exposure, with Hct typically rising until reaching a moderately elevated steady state after several weeks. However, adaptation to hypoxia involves a complex integration of oxygen transport mechanisms and cellular responses, which are not uniform across populations [3,4]. These variations are influenced by ancestry, genetic background, and environmental factors, making it more challenging to interpret Hb and Hct and distinguish between possible pathogenic changes and normal physiological responses [5,6].

Despite this, the World Health Organization (WHO) has defined reference values for anemia and polycytaemia in sea-level populations and altitude adjustment factors. For example, Hb levels exceeding 16 g/dL in women or 16.5 g/dL in men and Hct values exceeding 48% in women or 49% in men are indicators of suspicion according to the WHO diagnostic criteria for polycythemia vera (PV) [7]. Conversely, mild anemia is defined by Hb levels between 11–12 g/dL in women and 11–13 g/dL in men, whereas severe anemia corresponds to Hb levels below 8 g/dL in both sexes [8]. At moderate altitudes, it is recommended to subtract 0.2 g/dL Hb values from Hb values starting at 1000 m above sea level (m.a.s.l.) and 0.5 g/dL Hb values from 1500 m.a.s.l., with progressively greater adjustments required for populations residing at higher elevations [9].

The applicability of these reference values in high-altitude populations is controversial. A recent meta-analysis evaluating different populations worldwide reported that residents of the Andean Plateaus in South America show the greatest increase in Hb concentration (1 g/dL per 1000 m.a.s.l.). In contrast, in other regions such as the Tibetan Plateau and the Ethiopian Highlands, this increase was smaller (0.6 g/dL per 1000 m.a.s.l.) [5]. Moreover, current methods for adjusting Hb values according to altitude have significant limitations. The correction factors often cited are based on studies conducted in children (with an average age of 6 to 12 years) residing between 1200 and 3000 m.a.s.l., derived through curvilinear adjustments [10]. However, these values may not be appropriate for adults, since Hb levels in children are lower than those in adults, and there are sex- and age-related differences. For altitudes above 3000 m, reference values are derived from studies involving native high-altitude populations. This underscores the need to assess whether such adjustments are applicable to other populations exposed to similar conditions [5]. The corrected values may be underestimated for altitudes below 2000 m and overestimated for those above 3000 m. Additionally, one limitation of these studies is that they may include individuals who were born at lower altitudes but later migrated to higher elevations [6].

Defining population-specific reference values for Hb and Hct is crucial in clinical and diagnostic contexts. Each laboratory should establish reference ranges based on the demographic and geographic characteristics of the population it serves to minimize misinterpretation of results. This need is particularly relevant in countries with broad altitudinal variability, such as Colombia, where Hb and Hct values can differ substantially. Colombia has a highly diverse topography, with populations ranging from sea level to above 3000 m. Nearly 17 million people, almost one-third of the population, are estimated to reside between 2500 and 3000 m above sea level [11], where Hb and Hct values can differ markedly from those of low-altitude populations.

Although altitude significantly affects Hb and Hct levels, altitude-adjusted reference values for various regions of Colombia have not been established. This gap may contribute to misdiagnoses of anemia or polycythemia. Conventionally, reference values are determined using the direct method, which selects and analyzes samples from healthy individuals. However, this method is limited by selection bias, high costs, and challenges in generalizing results to heterogeneous populations. The application of Big Data and indirect methods has emerged as a promising alternative for estimating population reference values from large-scale routine laboratory data. Among these, refineR is an indirect algorithm recently validated by Ammer et al. (2021) [12]. RefineR employs an inverse modeling strategy and BoxCox transformations to extract non-pathological distributions from real-world datasets. Extensive simulations have demonstrated that refineR provides greater accuracy than other indirect approaches and even traditional direct methods with limited sample sizes, thereby offering a robust statistical basis for altitude-adjusted reference intervals. Accordingly, this study seeks to establish Hb and Hct reference values for the adult Colombian population, stratified by sex, age, and altitude, through the analysis of more than 670,000 patient records.

## 2. Materials and Methods

### 2.1. Study Design and Population

A cross-sectional study with retrospective data collection was conducted between January 2022 and December 2024. The source of information was the clinical laboratory system of Clínica Colsanitas, Colombia, which consolidates outpatient records from multiple healthcare centers nationwide, including Bogotá, Medellín, Cali, Bucaramanga, Barranquilla, and Armenia, among others. Data extraction was performed on 3 December 2024.

Patients older than 18 years were included. For women, two age groups (18–50 and >50 years) were defined to differentiate between presumably premenopausal and postmenopausal populations. In Latin America, the mean age at menopause is approximately 49.1 years; therefore, this threshold was selected for stratification [13]. The Hb (g/dL) and Hct (%) values were recorded from the date closest to the start of the study period and were measured via Sysmex hematology analyzers XN-1000, XN-550, and XP-300, (Sysmex Corporation, Kobe, Japan), which employs direct current detection and hydrodynamic focusing for RBC counting and non-cyanide Sodium Lauryl Sulfate method for Hb determination. The additional demographic variables collected included sex, age, place of residence, altitude, and reference health center.

To avoid overrepresentation, repeated test results within the same calendar year were excluded. Eligible patients were identified, and duplicate records were merged using medical record numbers as the primary criterion. Exclusion criteria were applied to remove records from hospital-based and nonhospital-based care (emergency visits, home care, and special programs) and patients outside the defined age range. Additionally, clinical cohorts with conditions that significantly alter hematological parameters, such as pregnant women, patients with cardiovascular disease, cancer, chronic kidney disease, or chronic obstructive pulmonary disease (COPD), were excluded.

The database was stratified by sex, age group, and altitude of residence and grouped into three categories: 0–1100 m, 1100–2000 m, and 2000–3000 m above sea level. This stratification allowed estimation of reference intervals for subgroups with distinct physiological and environmental characteristics.

### 2.2. Estimation of Reference Intervals

Statistical analysis focused on estimating reference intervals via a multistep approach, including data transformation, assessment of normality, outlier detection, and Gaussian model fitting. Box–Cox transformation was applied independently to each subgroup to stabilize variance and approximate normality. The lambda (λ) parameter was estimated through log-likelihood maximization; when λ equaled zero, a logarithmic transformation was used; otherwise, the general Box–Cox formula was applied.

The normality of the transformed data was evaluated via Kolmogorov–Smirnov, Lilliefor, and quantile–quantile (QQ) plots. A dataset was normally distributed when both statistical tests yielded *p* values > 0.05. Outlier detection was performed on the basis of the observed distribution. Tukey’s criterion was applied for normally distributed data, whereas for nonnormal data, the robust principal component method developed by Filzmoser et al. [13] was used.

**Figure 1:**
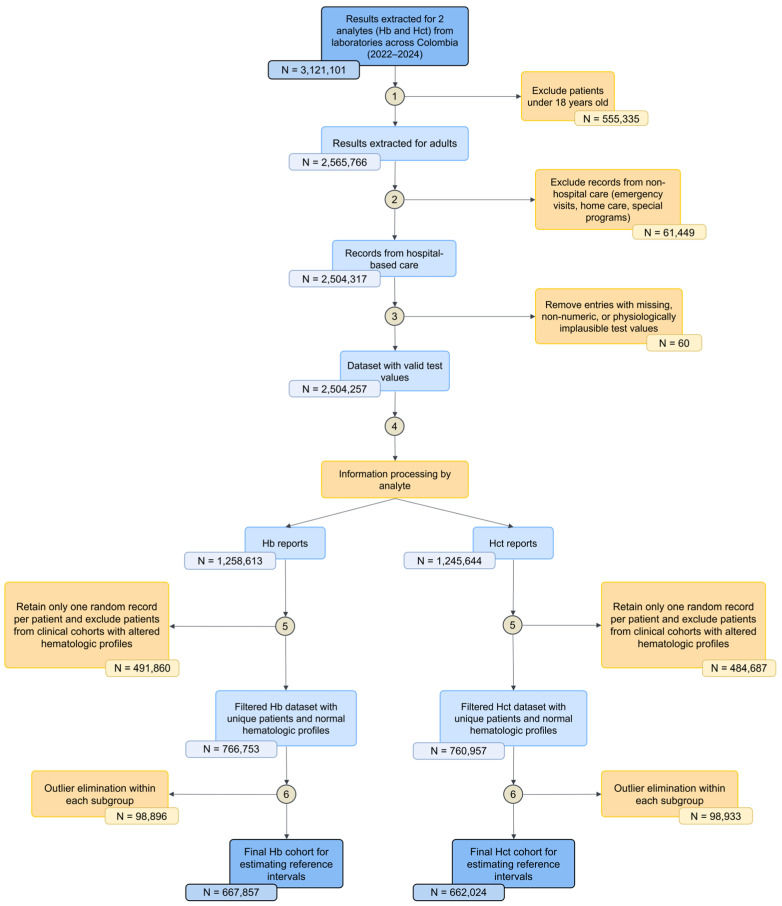
Study workflow diagram. Blue boxes represent datasets retained during the processing pipeline, while yellow boxes indicate exclusion criteria applied at each step. Circles denote sequential processing stages. Hb: hemoglobin; Hct: hematocrit.

Reference intervals were estimated via the refineR statistical package (v1.6.2) developed by Ammer et al. in 2021 [12]. This method applies an inverse, unbiased modeling approach to identify and isolate the non-pathological distribution from unlabeled, real-world data. To handle the inherent skewness of hemoglobin and hematocrit, the algorithm incorporates a Box–Cox transformation during the fitting process, utilizing a maximum likelihood cost function to determine the model that best explains the central healthy population while accounting for pathological contamination. Finally, an inverse Box–Cox transformation was applied to express the intervals in their original units, using a logarithmic transformation when λ = 0 and the general formula otherwise. Figure 1 illustrates the workflow applied to harmonize the Hb and Hct reference intervals, leveraging the potential of big data.

Additionally, analysis of variance (ANOVA) was conducted to assess whether significant differences existed in Hb and Hct levels across the five-year age strata within each subgroup. This analysis was performed on women aged 18–50 years, whereas it covered men aged 18–64 years. This step allowed us to determine whether reference intervals should be estimated by age subgroup or as a single combined interval.

## 3. Results

A total of 3,121,101 Hb and Hct test records from 2022 to 2024 were analyzed. After a rigorous data-cleaning process, we excluded records corresponding to hospital admissions, invalid ages, duplicates, non-numeric results, and patients from specific clinical cohorts. As a result, 667,857 unique hemoglobin and 662,024 hematocrit records were obtained, corresponding to apparently healthy adult patients, of whom 64% were women. The median age was 37 years (interquartile range [IQR]: 28–49), with similar values in women (36; IQR: 28–48) and men (38; IQR: 29–50) (Table 1).

**Figure 2:**
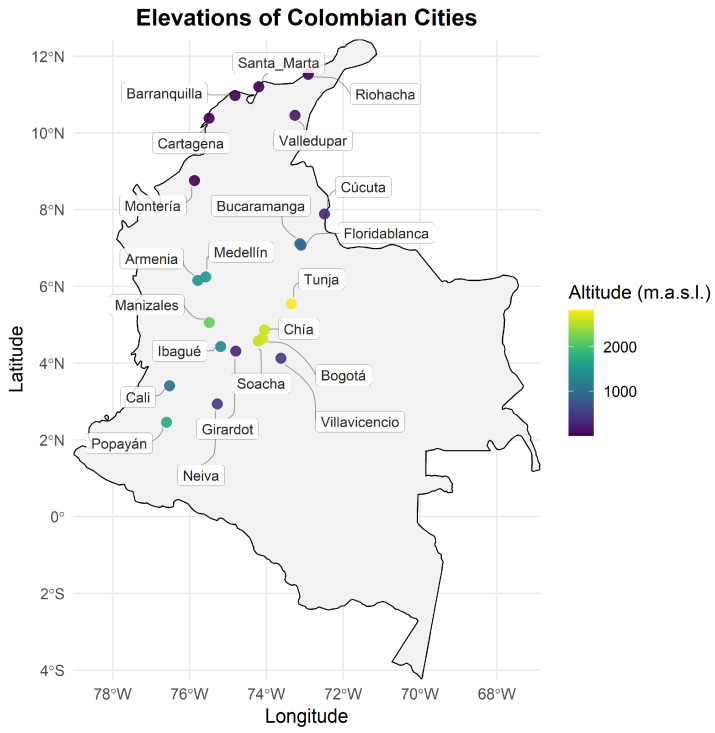
Distribution of the elevation of Colombian cities.

For the analysis of variables, records were grouped according to the altitude of the municipality of residence into three categories: low altitude (<1100 m a.s.l.), intermediate altitude (1100–2000 m a.s.l.), and high altitude (2000–3000 m a.s.l.). Detailed preprocessing results, including Box–Cox transformation parameters, normality tests, and outlier detection for each subgroup, are provided in the Supporting Information (Tables S1 and S2). Geographically, most participants resided in Bogotá (54.6%), which is consistent with the high proportion of individuals from high-altitude areas (57.6%) in the cohort, including municipalities such as Soacha, Chía, Tunja, and Manizales. The next most common locations were Cali (12.9%), the Central East region (12.5%), Bucaramanga (7.5%), Medellín (7.1%), and Barranquilla (5.5%). Overall, 31.3% of the participants were from low-altitude areas (including Cali, Barranquilla, Cartagena, Santa Marta, Montería, Valledupar, Villavicencio, and Bucaramanga), and 11.1% were from intermediate-altitude areas (including Medellín, Ibagué, Armenia, and Popayán) (Figure 2).

ANOVA revealed statistically significant differences in Hb and Htc levels across the evaluated five-year age groups (*p* < 0.05) (Tables S3 and S4). However, when assessing the magnitude of these differences, it was determined that they were not clinically relevant. In other words, although the mean values varied slightly among age groups, these changes remained within the expected physiological ranges and did not imply functional alterations or a differential diagnosis. Therefore, the observed statistical significance does not translate into a clinical need to stratify reference intervals by five-year age groups in all cases.

## 4. Reference Intervals for Hemoglobin and Hematocrit

**Figure 3:**
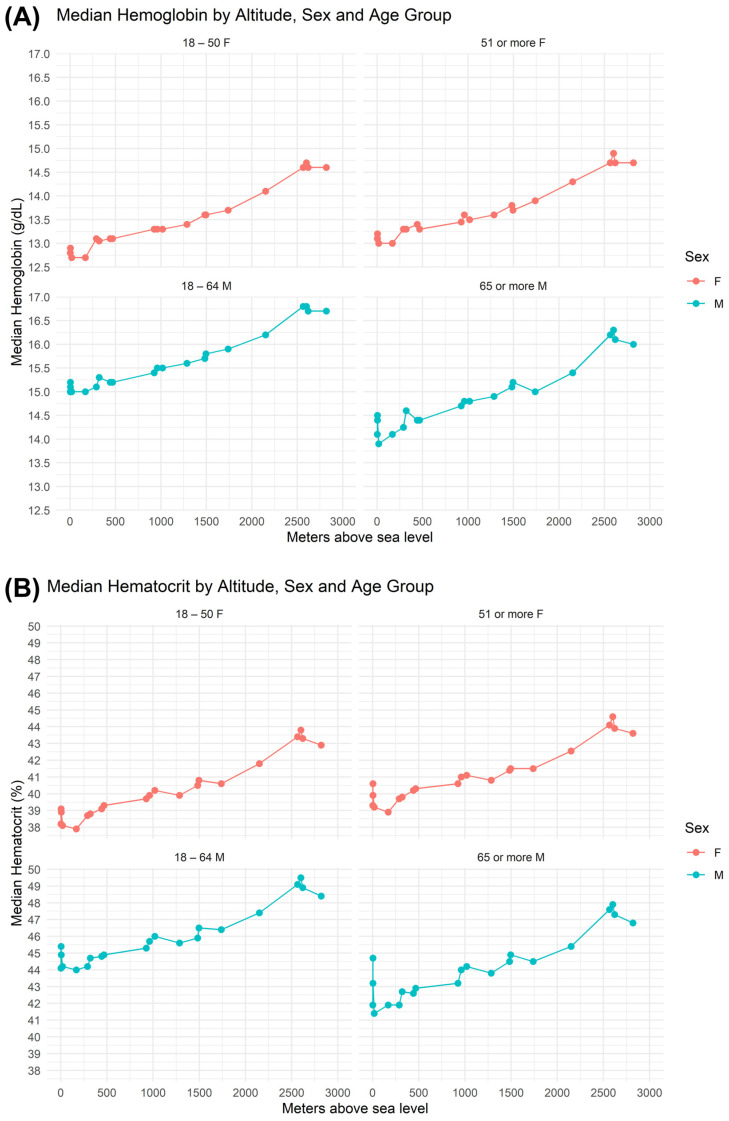
Distribution of (**A**) hemoglobin levels and (**B**) hematocrit levels by sex, altitude and age group.

The Hb and Hct reference intervals showed systematic variation according to sex, age, and residential altitude, with an ascending pattern at higher elevations across all the subgroups. Overall, men presented higher mean values than women did, and older adults presented greater interindividual variability, particularly at high altitudes (Figure 3).

For women aged 18–50 years, the hemoglobin reference intervals increased from 11.9–13.3 g/dL at low altitudes to 12.8–16.3 g/dL at high altitudes, with the median increasing from 13.2 g/dL to 14.6 g/dL. For men aged 18–64 years, the intervals increased from 15.1–16.3 g/dL to 16.3–17.3 g/dL, and the median increased from 15.3 g/dL to 16.7 g/dL. At intermediate altitudes, men presented a slight decrease in the midpoint of the reference interval to 15.57 g/dL, which may be related to demographic or methodological factors that require further investigation (see Table 2).

In individuals over the age of 64 years for men and 50 years for women, the increasing trend with altitude persisted. In women, reference intervals ranged from 13.2–14.2 g/dL (<1100 m.a.s.l.) to 14–14.9 g/dL at high altitude, whereas in men, the value ranged from 12.3–16.9 g/dL at low altitude to 13.9–18.6 g/dL at high altitude, and the median value climbed from 14.5 g/dL to 16.1 g/dL. This group also exhibited greater variability, as reflected by the wider interquartile range (15.3–16.9 g/dL).

Hematocrit values mirrored the altitude-associated pattern observed in hemoglobin. Among women aged 18–50 years, the median increased from 39.5% at low altitude to 43.3% at high altitude, accompanied by an expansion of the reference intervals from 34.5% to 45.1% to 38.4% to 46.7%. Among women older than 50 years, the median hematocrit reached 43.9% at high altitudes, within a reference range of 39.1–47.4%. Similarly, men aged 18–64 years presented an increase in the median hematocrit from 45.3% to 48.9% and a widening of the reference intervals from 39.8% to 49.0% to 43.5–53.7%. The greatest variability was observed among men older than 64 years, whose hematocrit values ranged from 39.8% to 54.5% at high altitudes, with a median of 47.3% (IQR 45.1–49.6%) (Table 2).

Application of conventional clinical cut-offs for erythrocytosis revealed a progressive increase in the proportion of individuals classified above the diagnostic threshold with rising altitude. Among women, the frequency of values exceeding the cut-off was zero at altitudes below 2000 m.a.s.l and remained low between 2000 and 3000 m (hemoglobin: 1.2–1.5%; hematocrit: ≤1.8%). In contrast, the effect was substantially greater in men. For those aged 18 to 64 years, the proportion exceeding the hemoglobin cut-off increased from 6.3% at altitudes below 1100 m to 16.1% at 1100–2000 m and 57.9% at 2000–3000 m, with a similar pattern observed for hematocrit (5.1%, 9.0%, and 47.9%, respectively). In men older than 64 years, the proportions reached 34.1% for hemoglobin and 29.9% for hematocrit at 2000–3000 m.

## 5. Discussion

The main objective of this study was to estimate reference values for Hb and Hct in the adult Colombian population via an indirect approach based on big data mining with nearly 3 million records. The analyzed cohort showed a predominance of women (64.5%), a trend consistent with findings from screening and preventive medicine studies in Latin America [14], attributable both to a slight female population predominance and to greater access to healthcare services, which in turn led to a greater number of laboratory tests. Geographically, more than half of the records (57.6%) came from cities located above 2000 m above sea level, including Bogotá, Manizales, Tunja, and Soacha, which may reflect the historical settlement pattern of the Colombian population in the inter-Andean valleys and plateaus of the three Andean mountain ranges, favored by geographic and climatic conditions that supported the development of large urban centers in these areas [15].

Based on the geographic distribution of the cohort, our findings indicate an upward trend in both parameters as the altitude of residence increased, affecting both women and men across all age groups. This pattern is consistent with the physiological mechanisms of adaptation to chronic hypoxia, which are widely described in the literature [3,6,16,17]. Several studies have demonstrated that increased Hb associated with altitude does not follow a linear pattern and varies significantly according to geographic region and ethnic origin [5,18]. The increase in Hb has been more pronounced in Andean populations than in other regions. This difference is partly attributed to the more recent human settlement of South America, which has resulted in a shorter period of genetic adaptation to high-altitude hypoxia in contrast to populations from other continents [3,16]. As a result, inhabitants of high-altitude regions in South America generally present higher Hb concentrations than those living at comparable altitudes in Asia or Africa. In this international comparative context, in the Sarawat highlands of Saudi Arabia (1500–3000 m.a.s.l.), mean Hb concentrations of 15.35–15.40 g/dL in men and 14.19–14.71 g/dL in women have been reported [19], and our Colombian population presented higher values of 13.10–16.60 g/dL in women and 16.39–17.36 g/dL in men at altitudes between 2000 and 3000 m.a.s.l.

The physiological response to altitude in our study follows the “Andean route” of adaptation described by Beall (2007) [20], characterized by an increase in Hb and Hct concentrations to compensate for hypoxia. However, the breadth of our reference intervals also reflects the complex genetic landscape of the Colombian population. According to recent data from the CÓDIGO consortium [21], the Colombian population is a tri-ethnic mosaic with varying proportions of European (50.6%), Indigenous American (32.8%), and African (16.7%) ancestry, organized into distinct regional clusters. This ancestral diversity, combined with significant internal migration between different thermal floors, explains the inter-individual variability observed in our study. To robustly capture this dynamic variability, we employed an indirect methodology; as demonstrated by Ammer et al. (2021) [12] and Zierk et al. (2015) [22], the use of large-scale clinical laboratory data is a validated approach for defining reference intervals that reflect real-world biological variation across age and sex groups. A key strength of the refineR algorithm is its ability to statistically isolate the non-pathological distribution, compensating for the absence of individual clinical data. Through inverse modeling, it filters pathological outliers, allowing the resulting intervals to more accurately reflect the central healthy population than traditional small-sample direct methods.

Our results are consistent with those reported in studies from the region. Arnaud et al. [23] reported Hb concentrations of 18.2 g/dL in Aymara populations and 15.8 g/dL in Quechua populations at 3600 m, whereas at altitudes below 450 m, Hb concentrations decreased to 14.8 g/dL and 13.2 g/dL, respectively. In Peru, research in adults residing in intermediate-altitude areas such as Arequipa (~2300 m) reported mean Hb values higher than those observed at sea level and similar to our cohort [18]. In contrast, in high-altitude Bolivian populations (>3000 m), even higher concentrations have been described, particularly in men, with means exceeding 16–18 g/dL [24]. Similarly, a study conducted in Medellín, Colombia, with more than 100,000 records, reported average hemoglobin and hematocrit values of 15.96 g/dL (±1.11) and 46.93% (±3.27), respectively, in men; in women, the values were 14.10 g/dL (±1.00) and 41.64% (±2.96) [25], very close to the intervals observed in our population at medium altitude.

In addition to the effect of altitude, a marked sex-related difference was observed, women between 18 and 50 years old had a median Hb that rose from 13.2 g/dL up to 1000 m of altitude to 14.6 g/dL between 2000 and 3000 m, and for those over 50 years old it went from 13.4 to 14.7 g/dL, without reaching the values of men who have a median of 15.3 gr/dL at less than one thousand meters of altitude and rises to 16.7 gr/dL when the altitude increases to 2000 to 3000 m; with systematically higher Hb and Hct values in men, women have mean levels approximately 12% lower than men. This difference is not explained by erythropoietin (EPO) levels or iron deficiency but rather by physiological mechanisms mediated by sex hormones [26]. In women, estrogen induces renal vasodilation, which improves oxygenation of the juxtaglomerular apparatus and attenuates the hypoxic signal that stimulates erythropoiesis. In contrast, in men, androgens exert a vasoconstrictive effect, enhancing EPO production and increasing red cell mass. Furthermore, owing to the same estrogen-mediated vasodilatory effect, women exhibit a greater proportion of red blood cells in the microvasculature, possibly due to larger capillary diameters (Fåhraeus effect), which contribute to efficient oxygenation with lower total circulating Hb [27]. These differences reflect a physiologically balanced state between sexes, with relevant metabolic and reproductive implications.

Our results revealed that, among men aged 18–64 years, the estimated mean hemoglobin value for the intermediate-altitude group was slightly lower than that for the low-altitude group, which is physiologically counterintuitive. The behavior of the refineR method can explain this observation. Unlike classical approaches that rely on raw averages or percentiles, refineR, as an inverse modeling approach, identifies the underlying distribution that best represents the presumably healthy population, implicitly excluding extreme values associated with pathological or atypical conditions [12]. The model may shift the distribution toward more conservative central values in contexts with greater biological or technical heterogeneity, such as the intermediate-altitude group, which combines data from cities with diverse population profiles. Furthermore, if a significant proportion of individuals have slightly reduced hemoglobin levels (due to causes such as subclinical iron deficiency or undiagnosed inflammatory diseases), refineR tends to shift the estimated interval to the left. While methodologically valid, these adjustments may yield mean or modal values that appear lower than those of more homogeneous or less dispersed groups. This behavior has previously been described in comparative validations with other methods, such as Hoffman or Kosmic, and it underscores the importance of interpreting indirect results considering dataset characteristics rather than solely on expected physiological assumptions. Although counterintuitive from a physiological perspective, this observation may be explained by greater data dispersion or asymmetric conservative values [28]. This methodological behavior is expected when working with mixed populations and heterogeneous subgroups, underscoring the need to interpret results based on the data structure rather than solely on simple population averages.

Older adults tend to have higher hemoglobin and hematocrit levels as altitude increases, but their values show greater variation. This variability may be due to several factors, including increased biological diversity, the presence of other medical conditions, and a reduced effectiveness of the body’s response to the production of red blood cells.

One contributing mechanism may be *inflammaging*, the chronic, low-grade inflammatory state associated with aging, which can reduce bone marrow responsiveness to erythropoietin and partially blunt the erythropoietic response [29]. The literature suggests that the effect of altitude on the Hb concentration persists even at advanced ages. For example, Alkhaldy et al. [19] compared adults residing at sea level and at 2270 m and reported that although hemoglobin levels tend to decrease slightly with age, the positive effect of altitude on these values remains robust in both sexes, even in individuals aged 80–100 years. However, they also reported that variability in Hb and Hct levels increases with age, reflecting a broader distribution of hematimetric results among older adults. Similarly, Patel et al. [30] reported that although mean hemoglobin decreases slightly with age, population variance progressively increases across age groups. This finding indicates that with aging, not only do hemoglobin values decrease, but their individual distribution range also increases, reflecting increasing biological heterogeneity.

Our study demonstrates a marked increase in the proportion of individuals exceeding conventional erythrocytosis cut-offs with rising altitude. Among men aged 18–64 years, the percentage above the hemoglobin threshold increased from 6.3% at <1100 m to 57.9% at 2000–3000 m, with a similar trend observed for hematocrit (5.1% to 47.9%). In men > 64 years, 34.1% exceeded the hemoglobin cut-off at 2000–3000 m. In contrast, women showed minimal impact of altitude, with proportions remaining ≤1.5% for hemoglobin and ≤1.8% for hematocrit even at higher elevations. According to the fifth edition of the WHO Classification of Haematolymphoid Tumours, PV is defined by hemoglobin > 16.5 g/dL or hematocrit > 49% in men, and hemoglobin > 16.0 g/dL or hematocrit > 48% in women [31]. When these fixed criteria are applied to populations residing at ≥2000 m.a.s.l., a substantial proportion of men may be flagged as having suspected PV, even though this likely reflects physiological adaptation to chronic hypoxia. Consequently, the use of non–altitude-adjusted cut-offs may markedly reduce diagnostic specificity, leading to unnecessary hematology referrals, molecular testing for JAK2 mutations, and potentially invasive procedures such as bone marrow biopsy. These results underscore the need for altitude-adjusted diagnostic frameworks to improve accuracy and avoid systematic overclassification in populations at moderate to high altitudes.

Finally, despite the robustness of our findings, this study has some limitations inherent to its design. Most importantly, no information was available regarding relevant clinical variables, such as smoking status, ethnicity, nutritional status, or micronutrient levels (iron, vitamin B12, and folate), all of which are known to influence hematological parameters. The absence of such detailed clinical data is directly related to the intrinsic challenges of data mining for establishing reference intervals from patient databases. These datasets include both physiological results and an unknown number of pathological values. Although the identification of physiological value distributions was based on predefined statistical assumptions, such as data normality after Box–Cox transformation and the predominance of normal results in the analyzed population, this approach does not allow detailed control of underlying clinical conditions. This represents, therefore, a methodological limitation inherent to retrospective studies based on large datasets.

## 6. Conclusions

In conclusion, this study represents a significant step toward the construction of hematological reference intervals tailored to Colombia’s geographic and population realities. Despite the limitations inherent to retrospective databases and the lack of individual clinical information, the findings obtained through robust statistical methods such as refineR make it possible to identify consistent patterns aligned with altitude-related physiology and the country’s biological diversity. Implementing these reference intervals, adapted to local contexts, could substantially improve the diagnostic accuracy of conditions such as anemia and polycythemia.

## Figures and Tables

**Table 1:** Distribution of hemoglobin and hematocrit values by sex and altitude in the study population.

Variable	All		Women		Men		All		Women		Men	
	*n* = 667,857	Hb (g/dL)	*n* = 427,460	Hb (g/dL)	*n* = 240,397	Hb (g/dL)	*n* = 662,024	Hct (%)	*n* = 425,702	Hct (%)	*n* = 236,322	Hct (%)
	*n* (%)	Median (IQR)	*n* (%)	Median (IQR)	*n* (%)	Median (IQR)	*n* (%)	Median (IQR)	*n* (%)	Median (IQR)	*n* (%)	Median (IQR)
Age (years) ^a^	37 (28–49)	14.7 (13.8–15.8)	36 (28–48)	14.1 (13.4–14.8)	38 (29–50)	16.1 (15.4–16.9)	37 (28–49)	43.7 (41.1–46.5)	37 (28–49)	42 (40–43.9)	38 (29–50)	47.4 (45.3–49.4)
Altitude (m.a.s.l)												
[0–1100)	209,174 (31.3)	13.8 (13.0–14.9)	131,299 (30.7)	13.2 (12.7–13.8)	77,875 (32.4)	15.3 (14.7–15.9)	207,616 (31.4)	41.4 (39.0–44.1)	130,838 (30.7)	39.7 (38.0–41.4)	76,778 (32.5)	45.2 (43.4–46.9)
[1100–2000)	73,817 (11.1)	14.2 (13.4–15.2)	47,951 (11.2)	13.6 (13.1–14.2)	25,866 (10.8)	15.7 (15.1–16.3)	72,630 (11.0)	42.1 (39.9–44.7)	47,182 (11.1)	40.6 (39.0–42.2)	25,448 (10.8)	46.0 (44.3–47.6)
[2000–3000]	384,866 (57.6)	15.2 (14.4–16.2)	248,210 (58.1)	14.6 (14.0–15.2)	136,656 (56.8)	16.7 (16.1–17.3)	381,778 (57.7)	45.0 (42.7–47.6)	247,682 (58.2)	43.4 (41.8–45.0)	134,096 (56.7)	48.8 (47.2–50.5)

^a^ Median (IQR). IQR: Interquartile range; Hb: hemoglobin; Hct: hematocrit; m.a.s.l: meters above sea level.

**Table 2:** Reference values for hemoglobin and hematocrit according to sex, age, and altitude.

Parameter	Gender	Age (Year)	Altitude (m.a.s.l)	RI	N (%)	Median	IQR	N Above Clinical Cut-Off (%)
Hb (g/dL)	F	18–50	[0–1100)	11.9–13.3	102,894 (15.4)	13.2	12.6–13.7	0 (0)
	F	18–50	[1100–2000)	12.0–14.6	37,168 (5.6)	13.6	13–14.1	0 (0)
	F	18–50	[2000–3000]	12.8–16.3	193,269 (28.9)	14.6	14–15.1	2251 (1.2)
	F	>50	[0–1100)	13.2–14.2	28,405 (4.2)	13.4	12.9–13.9	0 (0)
	F	>50	[1100–2000)	12.2–14.9	10,783 (1.6)	13.7	13.2–14.3	0 (0)
	F	>50	[2000–3000]	14.0–14.9	54,941 (8.2)	14.7	14.2–15.3	840 (1.5)
	M	18–64	[0–1100)	15.1–16.3	71,624 (10.7)	15.3	14.7–15.9	4520 (6.3)
	M	18–64	[1100–2000)	13.9–17.1	23,784 (3.6)	15.7	15.1–16.3	3823 (16.1)
	M	18–64	[2000–3000]	16.3–17.3	127,370 (19.1)	16.7	16.1–17.3	73,697 (57.9)
	M	>64	[0–1100)	12.3–16.9	6251 (0.9)	14.5	13.7–15.3	74 (1.2)
	M	>64	[1100–2000)	13.8–16.9	2082 (0.3)	15.0	14.3–15.7	99 (4.8)
	M	>64	[2000–3000]	13.9–18.6	9286 (1.4)	16.1	15.3–16.9	3168 (34.1)
Hct (%)	F	18–50	[0–1100)	34.5–45.1	102,155 (15.4)	39.5	37.8–41.2	0 (0)
	F	18–50	[1100–2000)	36.9–43.9	36,263 (5.4)	40.5	38.9–42.0	0 (0)
	F	18–50	[2000–3000]	38.4–46.7	191,177 (28.9)	43.3	41.6–44.8	0 (0)
	F	>50	[0–1100)	35.5–45.1	28,683 (4.3)	40.5	38.8–42.1	0 (0)
	F	>50	[1100–2000)	36.3–45.2	10,919 (1.6)	41.2	39.7–42.8	0 (0)
	F	>50	[2000–3000]	39.1–47.4	56,505 (8.5)	43.9	42.3–45.5	992 (1.8)
	M	18–64	[0–1100)	39.8–49.0	70,603 (10.6)	45.3	43.6–47.0	3569 (5.1)
	M	18–64	[1100–2000)	40.4–51.7	23,320 (3.5)	46.1	44.5–47.7	2101 (9.0)
	M	18–64	[2000–3000]	43.5–53.7	124,879 (18.9)	48.9	47.3–50.6	59,779 (47.9)
	M	>64	[0–1100)	34.9–49.6	6175 (0.9)	43.3	40.9–45.5	70 (1.1)
	M	>64	[1100–2000)	35.7–50.2	2128 (0.3)	44.4	42.2–46.4	88 (4.1)
	M	>64	[2000–3000]	39.8–54.5	9217 (1.4)	47.3	45.1–49.6	2754 (29.9)

M: males; F: females; RI: reference interval; IQR: interquartile range; Hb: hemoglobin; Hct: hematocrit; m.a.s.l: meters above sea level. Clinical cut-offs defined as: Hb > 16.0 g/dL (F), >16.5 g/dL (M); Hct > 48% (F), >49% (M).

## Data Availability

All relevant data are within the paper and its Supporting Information files. Direct access to the raw database is restricted due to ethical considerations.

## Supplementary Materials

The following supporting information can be downloaded at: https://www.mdpi.com/article/10.3390/medsci14010136/s1, Table S1. Data preprocessing summary for hemoglobin (Hb) subsets. Table S2. Data preprocessing summary for hematocrit (Hct) subsets. Table S3. ANOVA results for hemoglobin (Hb) by age group and altitude. Table S4. ANOVA results for hematocrit (Hct) by age group and altitude.
