# Supplementary material for: Reference Intervals for Hemoglobin and Hematocrit Adjusted for Altitude, Sex, and Age: A Big Data-Based Study in the Colombian Population
Source: Med Sci (Basel). 2026 Mar 14;14(1):136. doi: 10.3390/medsci14010136 (PMC13027793; doi:10.3390/medsci14010136)
Supplement: Supplementary file 1 [file medsci-14-00136-s001.zip › S1. Data Preprocessing Hb Subsets.pdf]

S1 Table. Data preprocessing summary for hemoglobin (Hb) subsets.

<table>
<tr><th colspan="4">Subset: 18 – 50 years (F) | Altitude: [0-1100) m.a.s.l</th></tr>
<tr>
<td>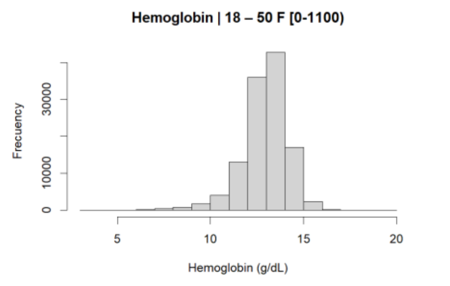
</td>
<td>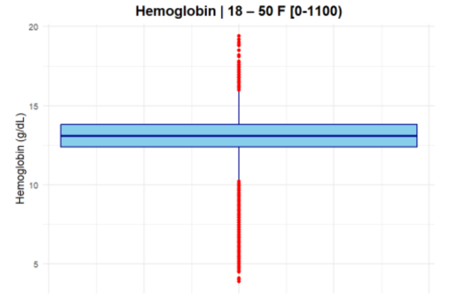
</td>
<td>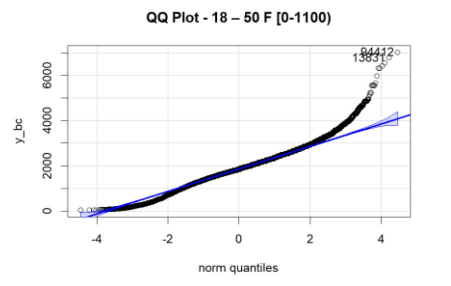
</td>
<td>N (original): 118,746
Lambda Box-Cox: 3.4
Kolmogorov-Smirnov p: 0
Lilliefors p: 0
Normal Distribution: No
Outliers (Hubert): 15,852</td>
</tr>
<tr>
<td>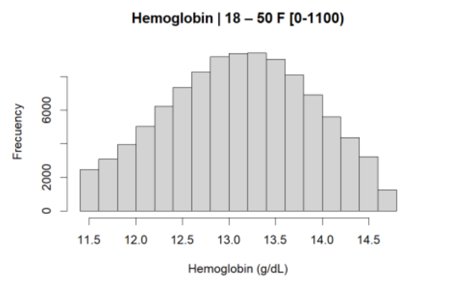
</td>
<td>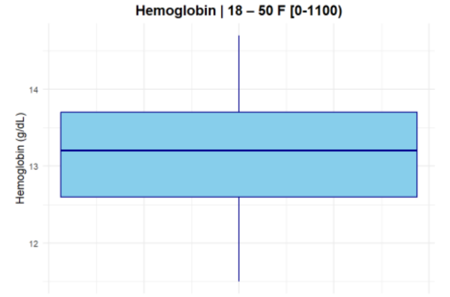
</td>
<td>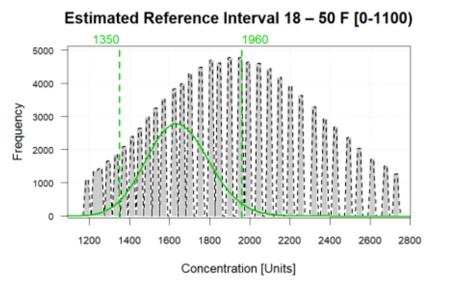
</td>
<td>N (final): 102,894

Reference Intervals
lower limit [2.5% perc]: 1,350
upper limit [97.5% perc]: 1,960
RI [g/dL]: 11.94 - 13.33</td>
</tr>
<tr><th colspan="4">Subset: 18 – 50 years (F) | Altitude: [1100-2000) m.a.s.l</th></tr>
<tr>
<td>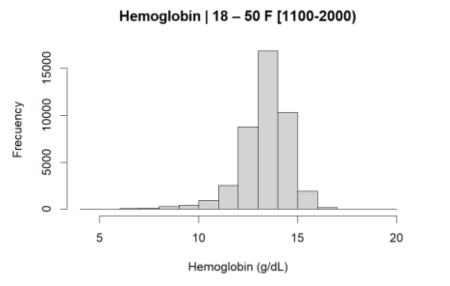
</td>
<td>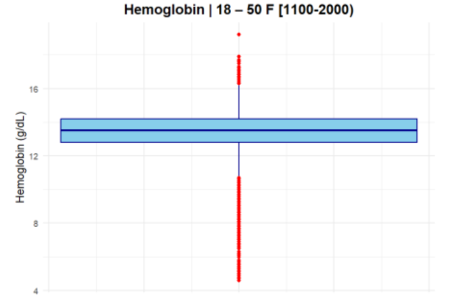
</td>
<td>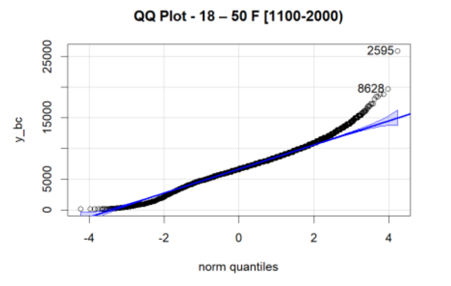
</td>
<td>N (original): 42,418
Lambda Box-Cox: 3.9
Kolmogorov-Smirnov p: 0
Lilliefors p: 0
Normal Distribution: No
Outliers (Hubert): 5,250</td>
</tr>
<tr>
<td>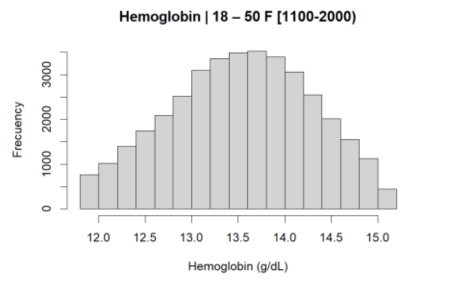
</td>
<td>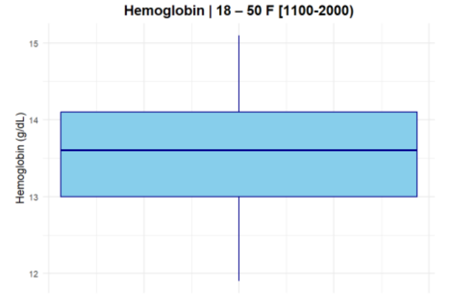
</td>
<td>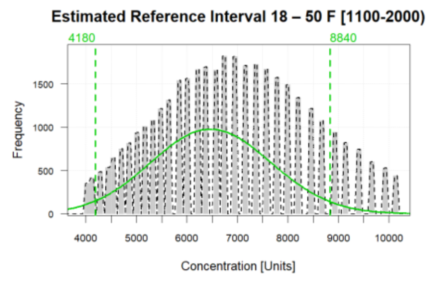
</td>
<td>N (final): 37,168

Reference Intervals
lower limit [2.5% perc]: 4,180
upper limit [97.5% perc]: 8,840
RI [g/dL]: 12.03 - 14.57</td>
</tr>
</table>

Subset: 18 – 50 years (F) | Altitude: [2000-3000] m.a.s.l

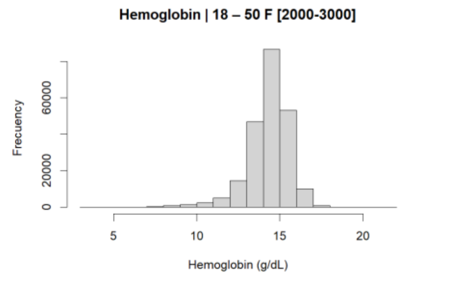

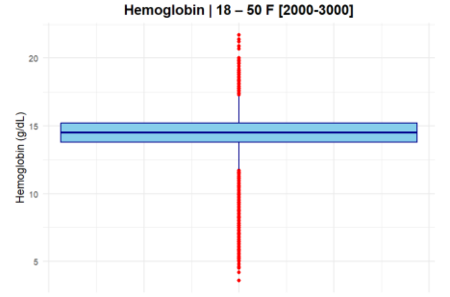

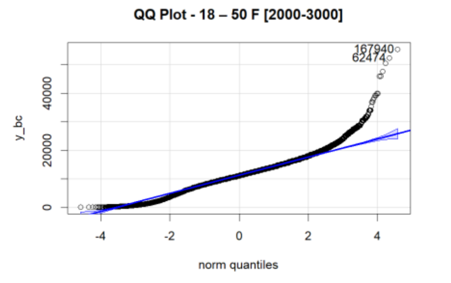

N (original): 222,320
Lambda Box-Cox: 4
Kolmogorov-Smirnov p: 0
Lilliefors p: 0
Normal Distribution: No
Outliers (Hubert): 29,051

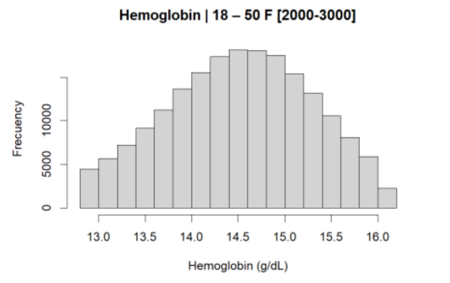

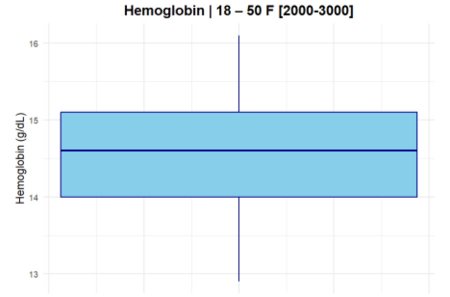

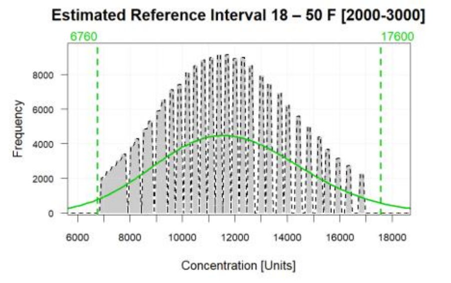

N (final): 193,269

Reference Intervals
lower limit [2.5% perc]: 6,760
upper limit [97.5% perc]: 17,600
RI [g/dL]: 12.82 - 16.28

Subset: >50 years (F) | Altitude: [0-1100) m.a.s.l

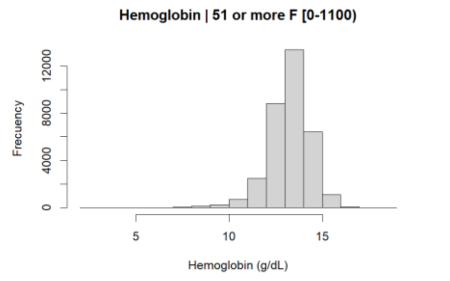

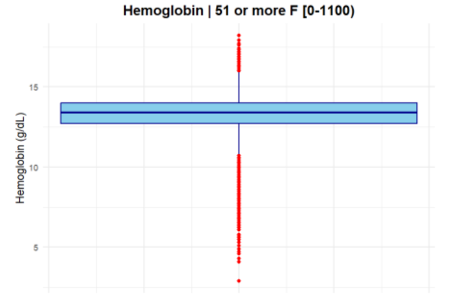

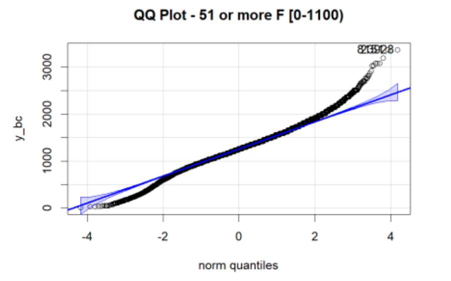

N (original): 33,508
Lambda Box-Cox: 3.2
Kolmogorov-Smirnov p: 0
Lilliefors p: 0
Normal Distribution: No
Outliers (Hubert): 5,103

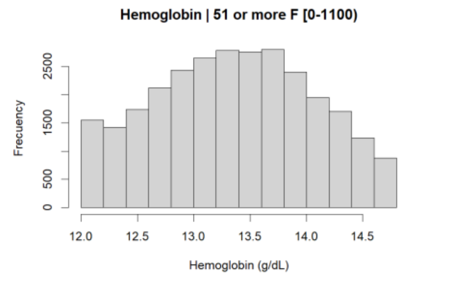

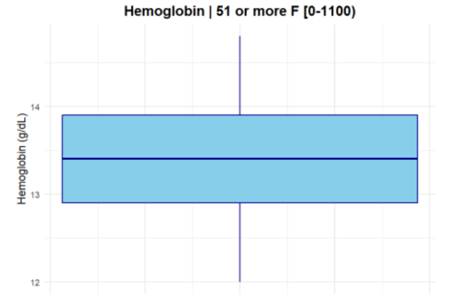

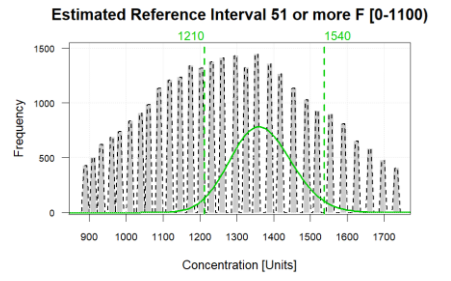

N (final): 28,405

Reference Intervals
lower limit [2.5% perc]: 1,210
upper limit [97.5% perc]: 1,540
RI [g/dL]: 13.23 - 14.25

Subset: >50 years (F) | Altitude: [1100-2000) m.a.s.l

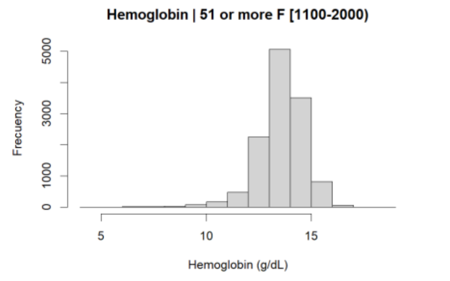

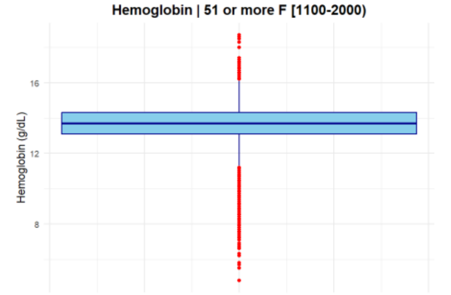

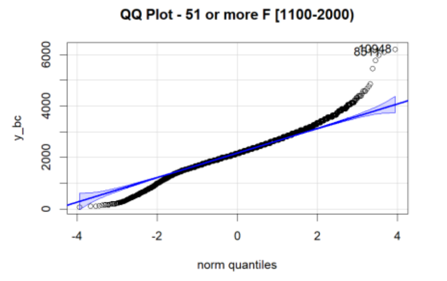

N (original): 12,525
Lambda Box-Cox: 3.4
Kolmogorov-Smirnov p: 0
Lilliefors p: 0
Normal Distribution: No
Outliers (Hubert): 1,742

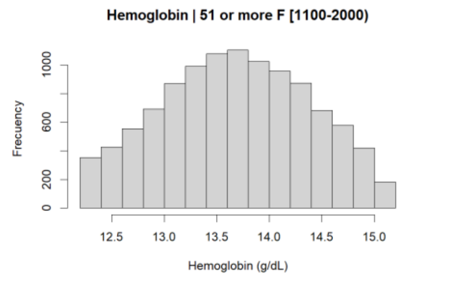

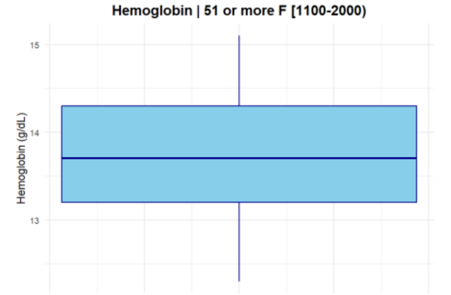

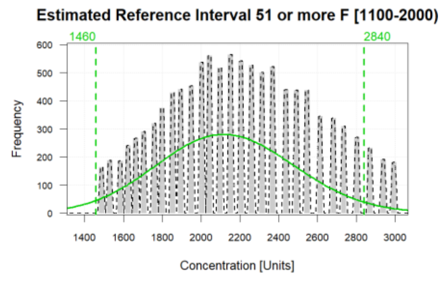

N (final): 10,783

Reference Intervals
lower limit [2.5% perc]: 1,460
upper limit [97.5% perc]: 2,840
RI [g/dL]: 12.21 - 14.86

Subset: >50 years (F) | Altitude: [2000-3000] m.a.s.l

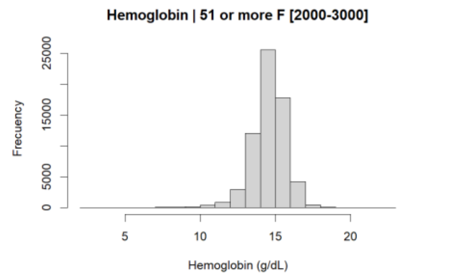

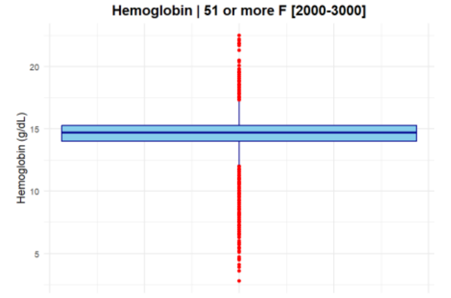

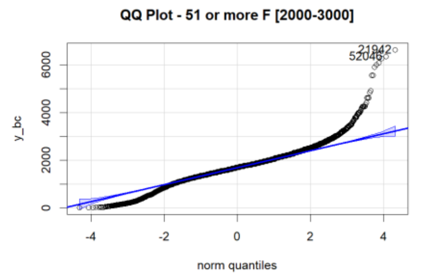

N (original): 64,940
Lambda Box-Cox: 3.2
Kolmogorov-Smirnov p: 0
Lilliefors p: 0
Normal Distribution: No
Outliers (Hubert): 9,999

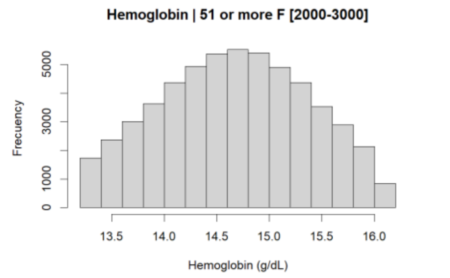

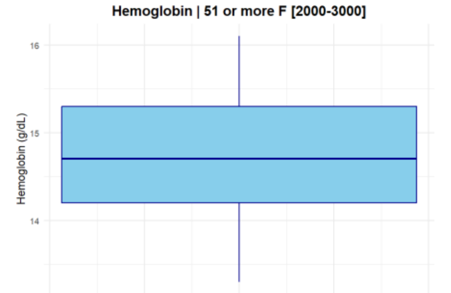

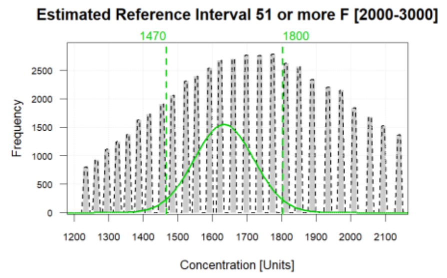

N (final): 54,941

Reference Intervals
lower limit [2.5% perc]: 1,470
upper limit [97.5% perc]: 1,800
RI [g/dL]: 14.04 - 14.98

Subset: 18 – 64 years (M) | Altitude: [0-1100) m.a.s.l

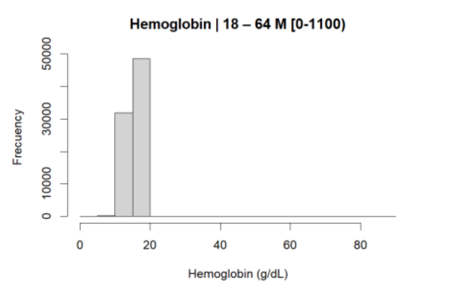

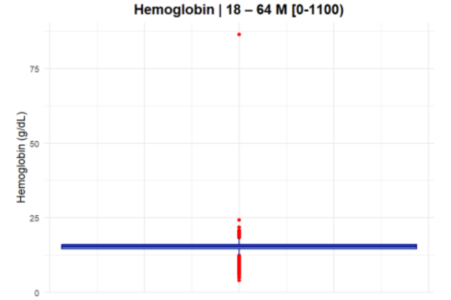

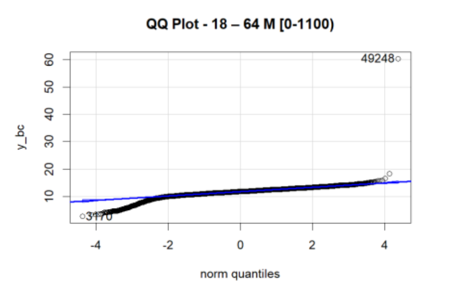

N (original): 80,736
Lambda Box-Cox: 0.9
Kolmogorov-Smirnov p: 0
Lilliefors p: 0
Normal Distribution: No
Outliers (Hubert): 9,112

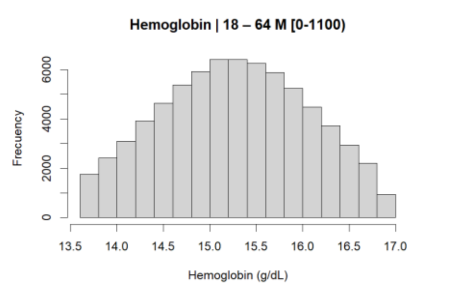

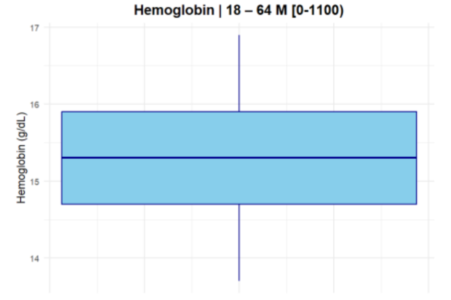

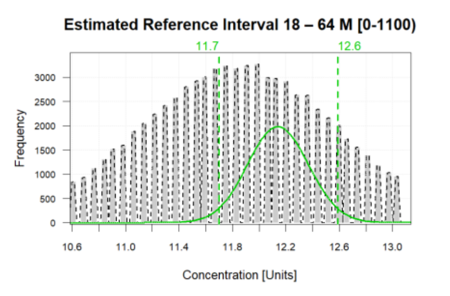

N (final): 71,624

Reference Intervals
lower limit [2.5% perc]: 11.7
upper limit [97.5% perc]: 12.6
RI [g/dL]: 15.13 - 16.30

Subset: 18 – 64 years (M) | Altitude: [1100-2000) m.a.s.l

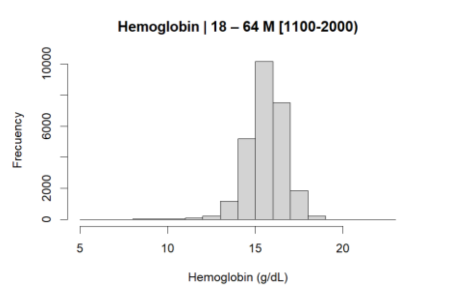

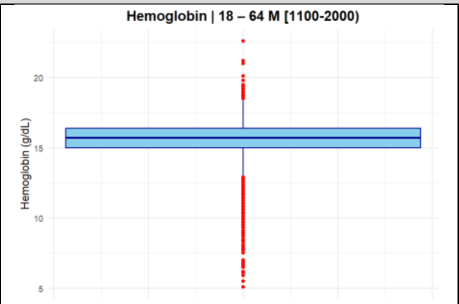

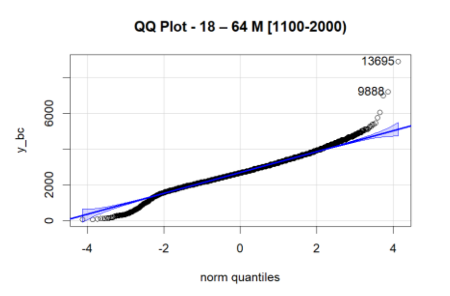

N (original): 26,557
Lambda Box-Cox: 3.3
Kolmogorov-Smirnov p: 0
Lilliefors p: 0
Normal Distribution: No
Outliers (Hubert): 2,773

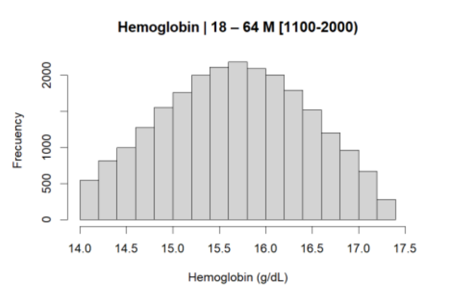

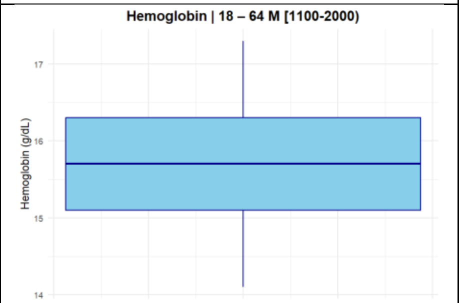

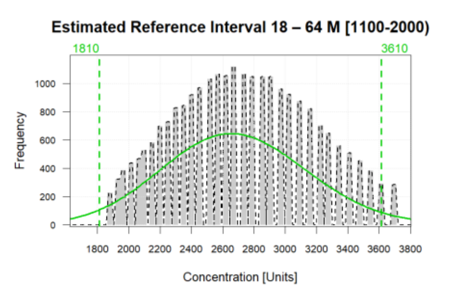

N (final): 23,784

Reference Intervals
lower limit [2.5% perc]: 1,810
upper limit [97.5% perc]: 3,610
RI [g/dL]: 13.95 - 17.19

<table>
<tr><th colspan="4">Subset: 18 – 64 years (M) | Altitude: [2000-3000] m.a.s.l</th></tr>
<tr>
<td>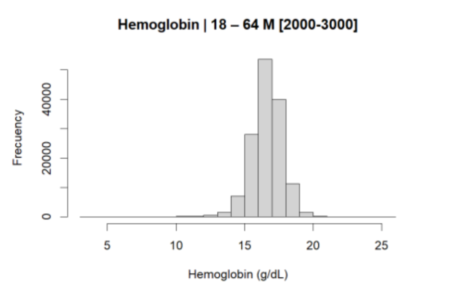</td>
<td>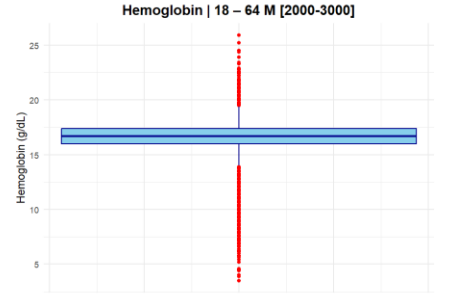</td>
<td>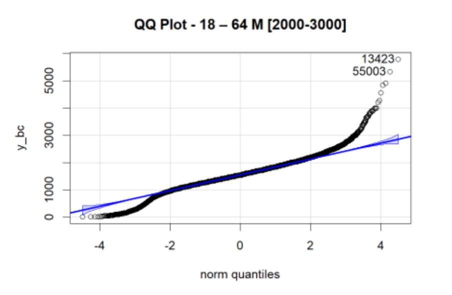</td>
<td>N (original): 144,440
Lambda Box-Cox: 3
Kolmogorov-Smirnov p: 0
Lilliefors p: 0
Normal Distribution: No
Outliers (Hubert): 17,070</td>
</tr>
<tr>
<td>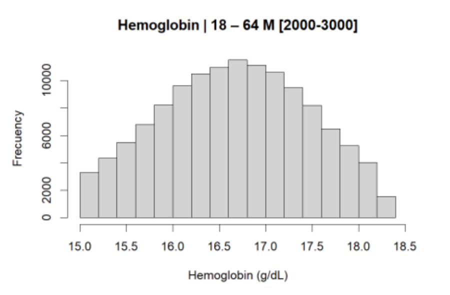</td>
<td>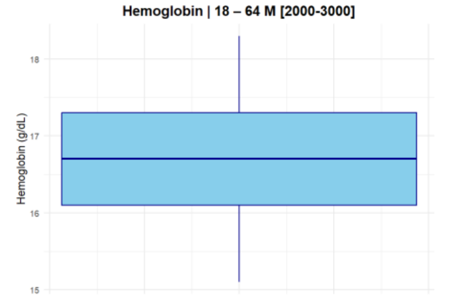</td>
<td>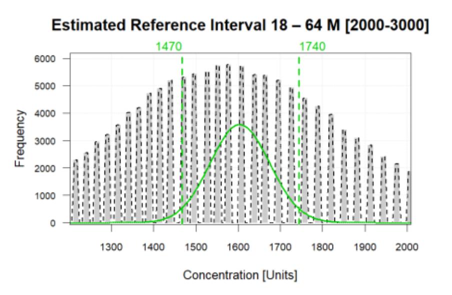</td>
<td>N (final): 127,370

Reference Intervals
lower limit [2.5% perc]: 1,470
upper limit [97.5% perc]: 1,740
RI [g/dL]: 16.39 - 17.36</td>
</tr>
<tr><th colspan="4">Subset: >64 years (M) | Altitude: [0-1100) m.a.s.l</th></tr>
<tr>
<td>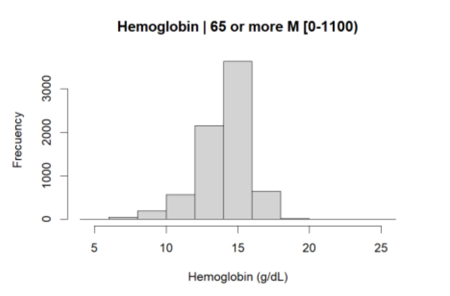</td>
<td>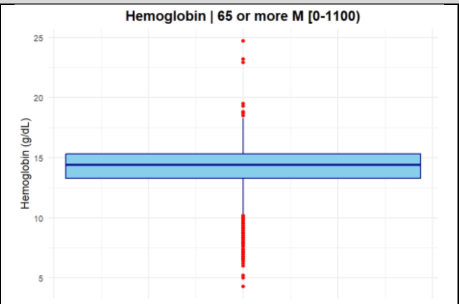</td>
<td>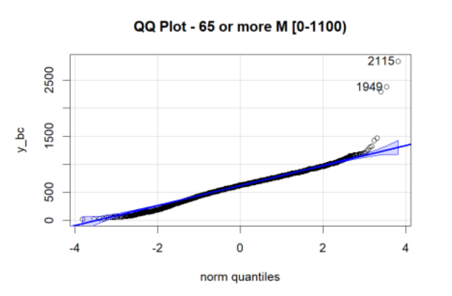</td>
<td>N (original): 7,260
Lambda Box-Cox: 2.8
Kolmogorov-Smirnov p: 0
Lilliefors p: 0
Normal Distribution: No
Outliers (Hubert): 1,009</td>
</tr>
<tr>
<td>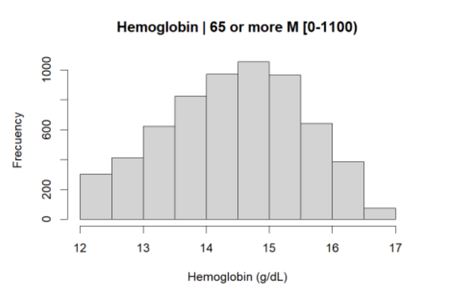</td>
<td>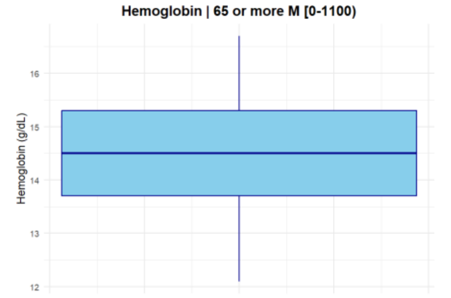</td>
<td>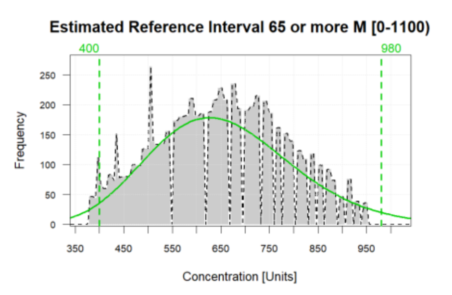</td>
<td>N (final): 6,251

Reference Intervals
lower limit [2.5% perc]: 400
upper limit [97.5% perc]: 980
RI [g/dL]: 12.28 - 16.91</td>
</tr>
</table>

Subset: >64 years (M) | Altitude: [1100-2000) m.a.s.l

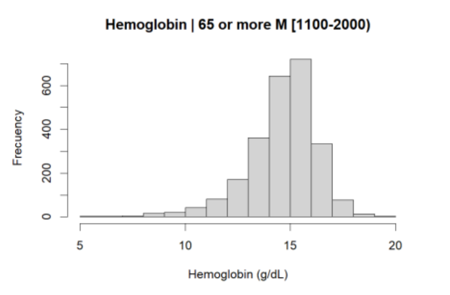

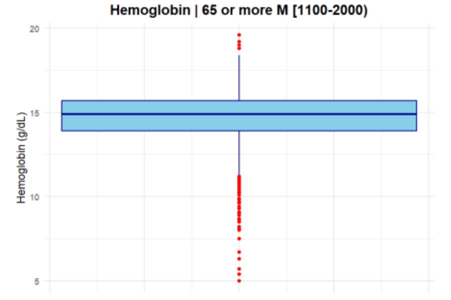

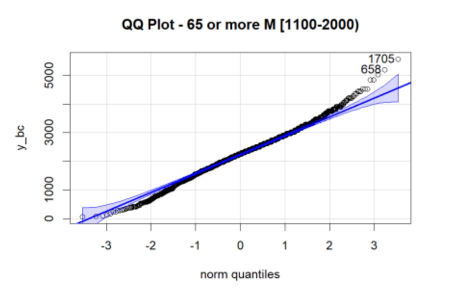

N (original): 2,489
Lambda Box-Cox: 3.3
Kolmogorov-Smirnov p: 0.0045
Lilliefors p: 0
Normal Distribution: No
Outliers (Hubert): 407

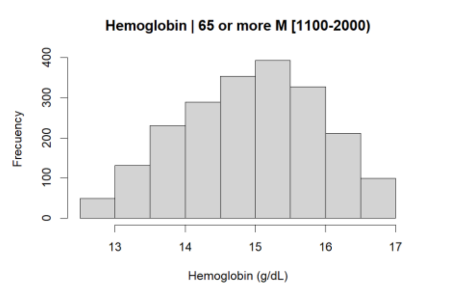

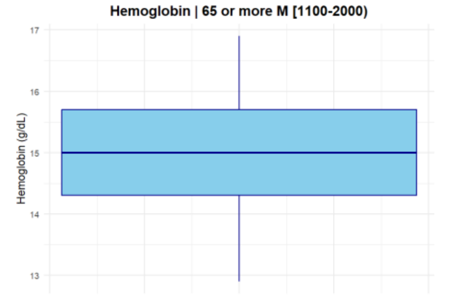

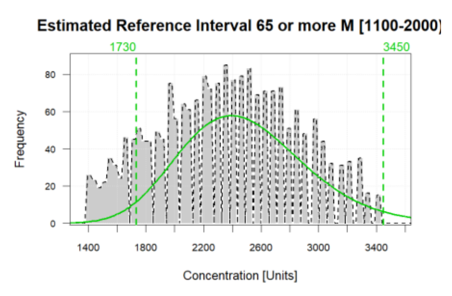

N (final): 2,082

Reference Intervals
lower limit [2.5% perc]: 1,730
upper limit [97.5% perc]: 3,450
RI [g/dL]: 13.76 - 16.95

Subset: >64 years (M) | Altitude: [2000-3000] m.a.s.l

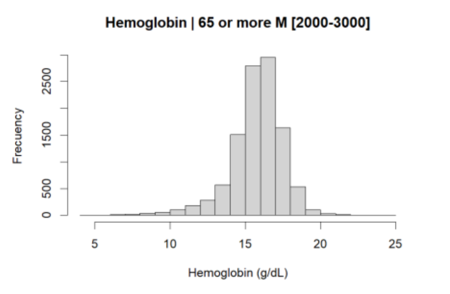

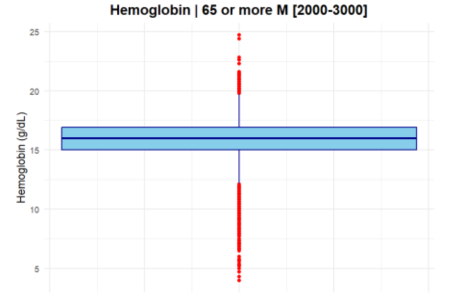

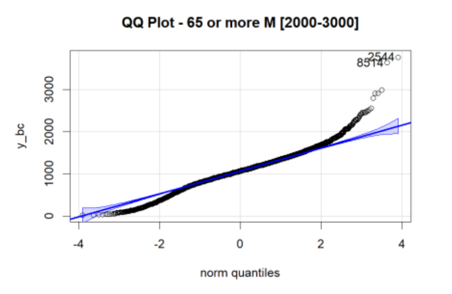

N (original): 10,814
Lambda Box-Cox: 2.9
Kolmogorov-Smirnov p: 0
Lilliefors p: 0
Normal Distribution: No
Outliers (Hubert): 1,528

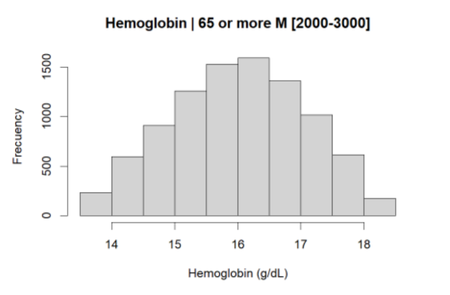

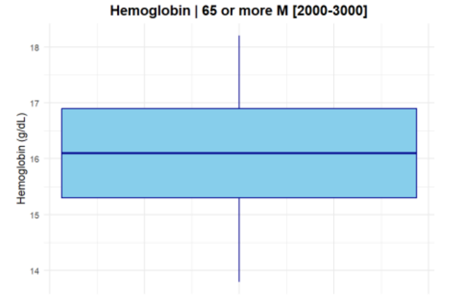

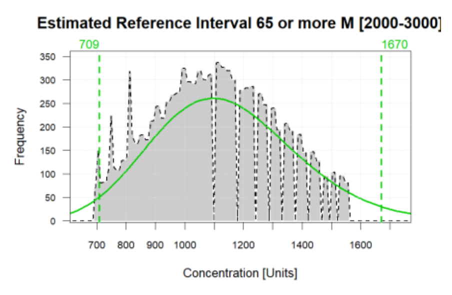

N (final): 9,286

Reference Intervals
lower limit [2.5% perc]: 709
upper limit [97.5% perc]: 1,670
RI [g/dL]: 13.88 - 18.65
