# Supplementary material for: Reference Intervals for Hemoglobin and Hematocrit Adjusted for Altitude, Sex, and Age: A Big Data-Based Study in the Colombian Population
Source: Med Sci (Basel). 2026 Mar 14;14(1):136. doi: 10.3390/medsci14010136 (PMC13027793; doi:10.3390/medsci14010136)
Supplement: Supplementary file 1 [file medsci-14-00136-s001.zip › S2. Data Preprocessing Hct Subsets.pdf]

S2 Table. Data preprocessing summary for hematocrit (Hct) subsets.

<table>
<tr><th colspan="4">Subset: 18 – 50 years (F) | Altitude: [0-1100) m.a.s.l</th></tr>
<tr>
<td>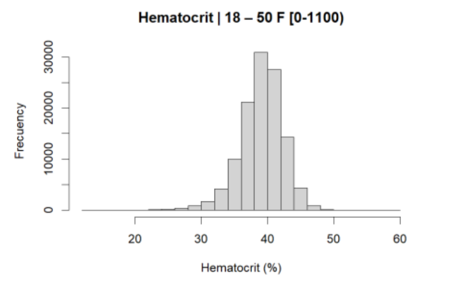
</td>
<td>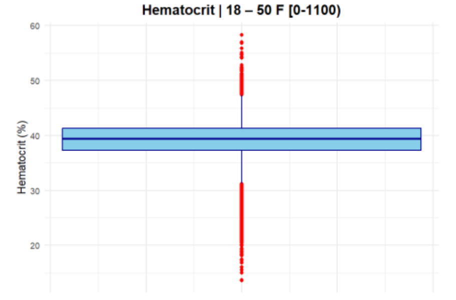
</td>
<td>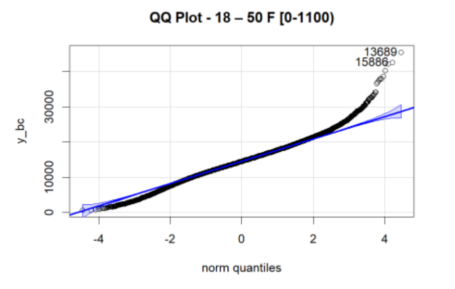
</td>
<td>N (original): 116,828
Lambda Box-Cox: 2.9
Kolmogorov-Smirnov p: 0
Lilliefors p: 0
Normal Distribution: No
Outliers (Hubert): 14,673</td>
</tr>
<tr>
<td>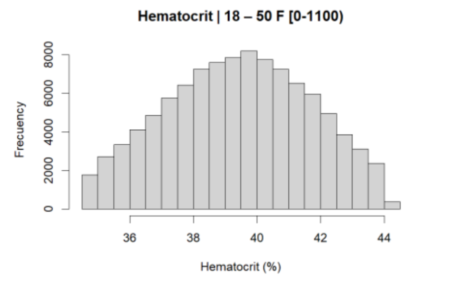
</td>
<td>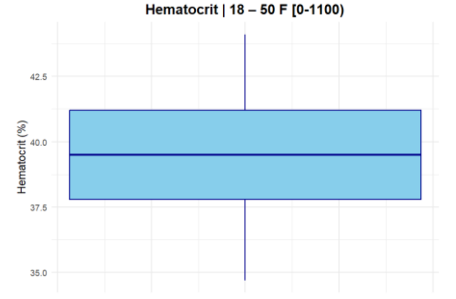
</td>
<td>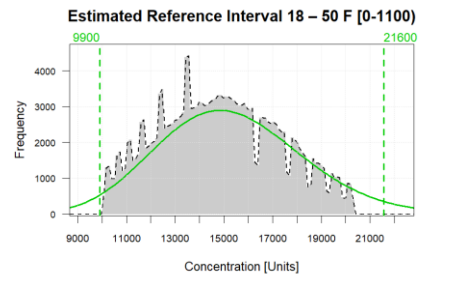
</td>
<td>N (final): 102,155

Reference Intervals
lower limit [2.5% perc]: 9,900
upper limit [97.5% perc]: 21,600
RI [%]: 34.46 - 45.07</td>
</tr>
<tr><th colspan="4">Subset: 18 – 50 years (F) | Altitude: [1100-2000) m.a.s.l</th></tr>
<tr>
<td>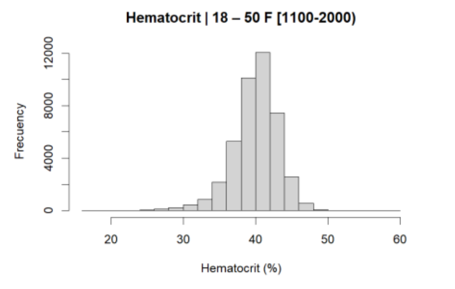
</td>
<td>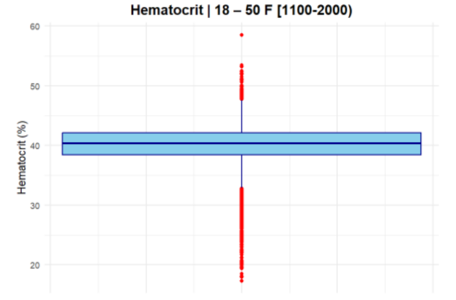
</td>
<td>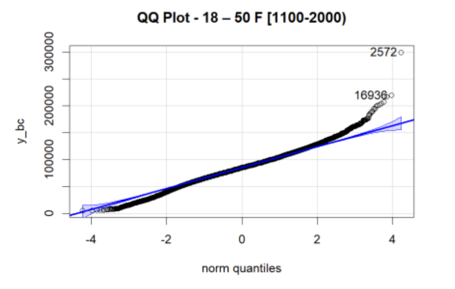
</td>
<td>N (original): 42,080
Lambda Box-Cox: 3.4
Kolmogorov-Smirnov p: 0
Lilliefors p: 0
Normal Distribution: No
Outliers (Hubert): 5,817</td>
</tr>
<tr>
<td>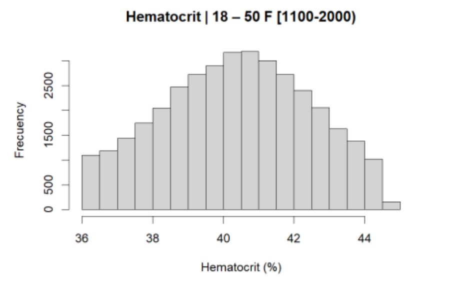
</td>
<td>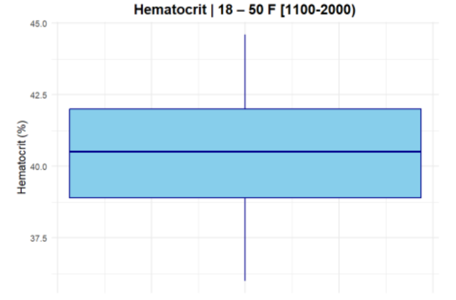
</td>
<td>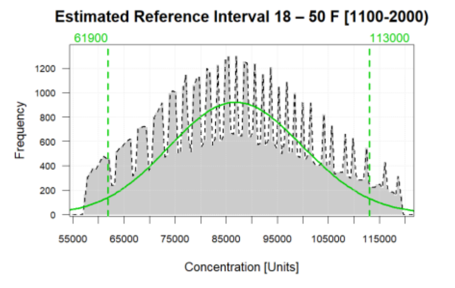
</td>
<td>N (final): 36,263

Reference Intervals
lower limit [2.5% perc]: 61,900
upper limit [97.5% perc]: 113,000
RI [%]: 36.78 - 43.91</td>
</tr>
</table>

Subset: 18 – 50 years (F) | Altitude: [2000-3000] m.a.s.l

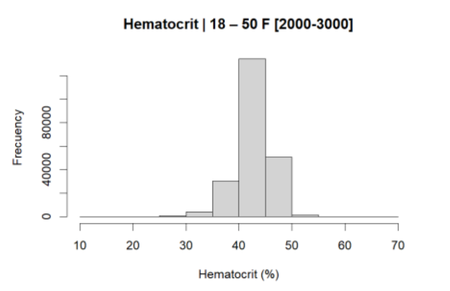

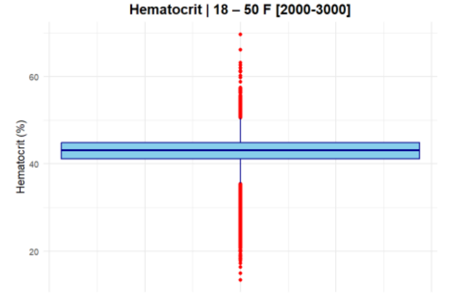

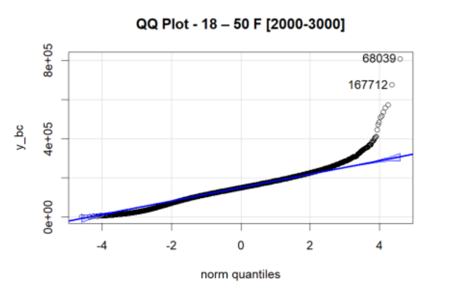

N (original): 221,655
Lambda Box-Cox: 3.5
Kolmogorov-Smirnov p: 0
Lilliefors p: 0
Normal Distribution: No
Outliers (Hubert): 30,478

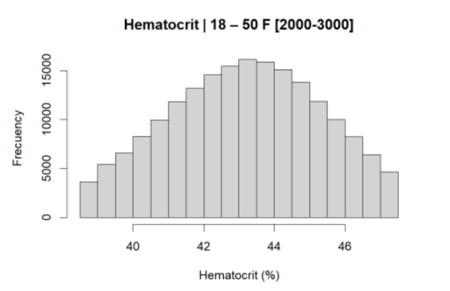

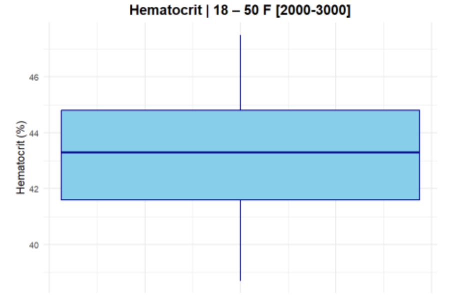

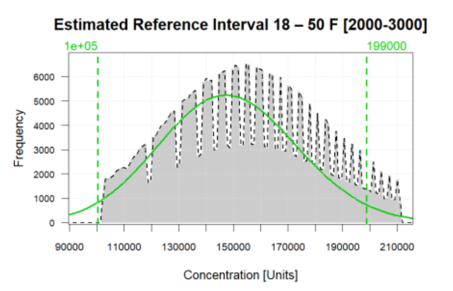

N (final): 191,177

Reference Intervals
lower limit [2.5% perc]: 100,000
upper limit [97.5% perc]: 199,000
RI [%]: 38.41 - 46.70

Subset: >50 years (F) | Altitude: [0-1100) m.a.s.l

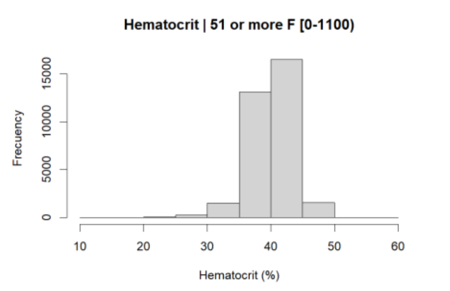

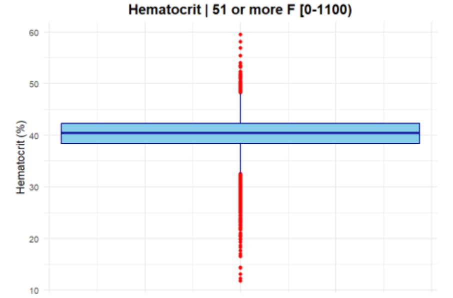

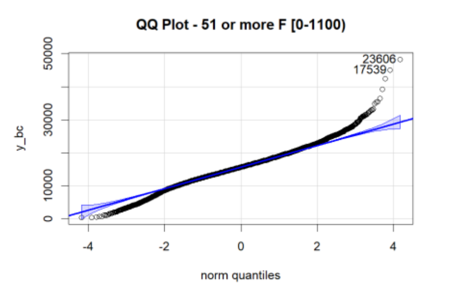

N (original): 33,037
Lambda Box-Cox: 2.9
Kolmogorov-Smirnov p: 0
Lilliefors p: 0
Normal Distribution: No
Outliers (Hubert): 4,354

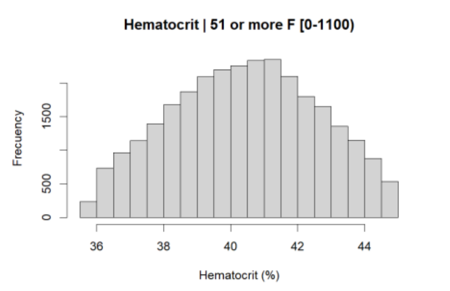

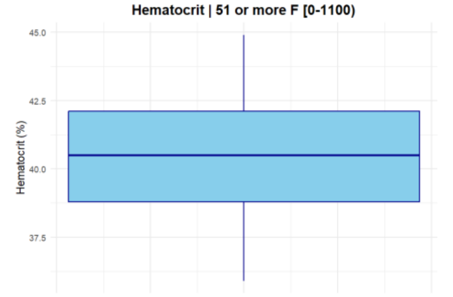

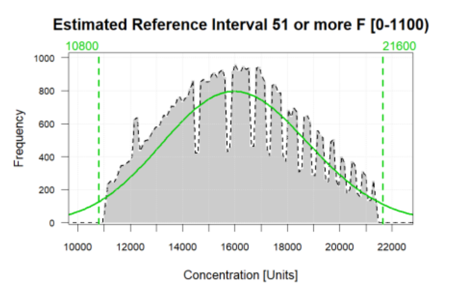

N (final): 28,683

Reference Intervals
lower limit [2.5% perc]: 10,800
upper limit [97.5% perc]: 21,600
RI [%]: 35.50 - 45.13

Subset: >50 years (F) | Altitude: [1100-2000) m.a.s.l

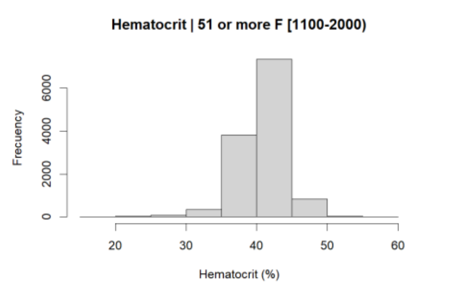

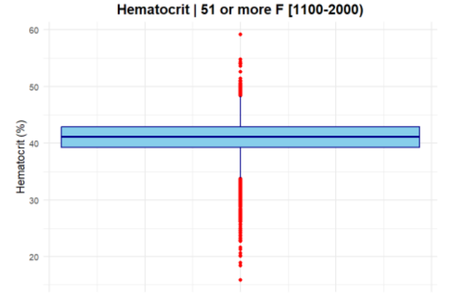

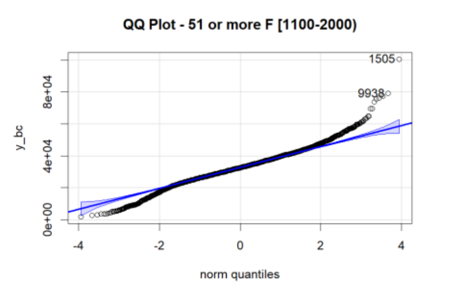

N (original): 12,475
Lambda Box-Cox: 3.1
Kolmogorov-Smirnov p: 0
Lilliefors p: 0
Normal Distribution: No
Outliers (Hubert): 1,556

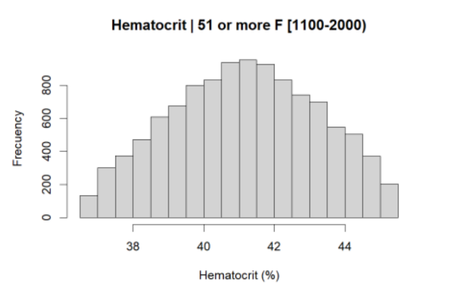

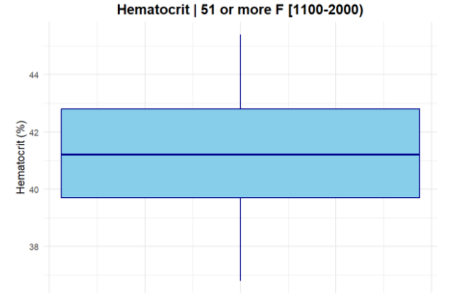

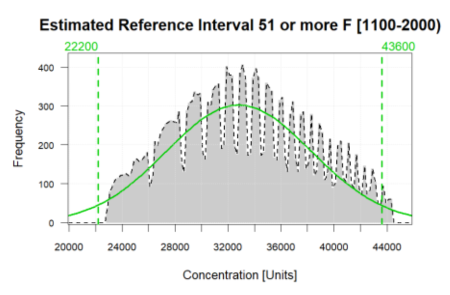

N (final): 10,919

Reference Intervals
lower limit [2.5% perc]: 22,200
upper limit [97.5% perc]: 43,600
RI [%]: 36.35 - 45.21

Subset: >50 years (F) | Altitude: [2000-3000] m.a.s.l

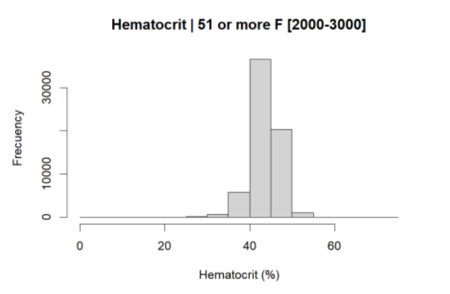

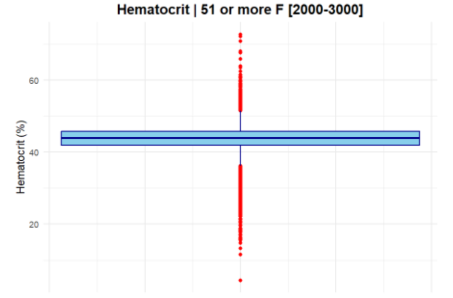

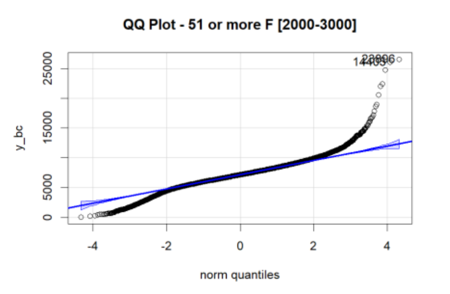

N (original): 64,701
Lambda Box-Cox: 2.6
Kolmogorov-Smirnov p: 0
Lilliefors p: 0
Normal Distribution: No
Outliers (Hubert): 8,196

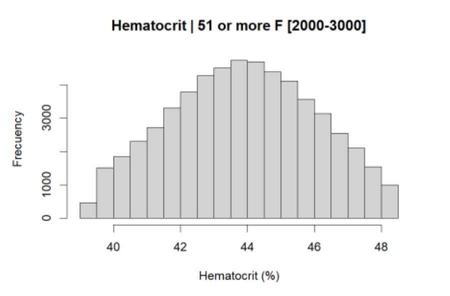

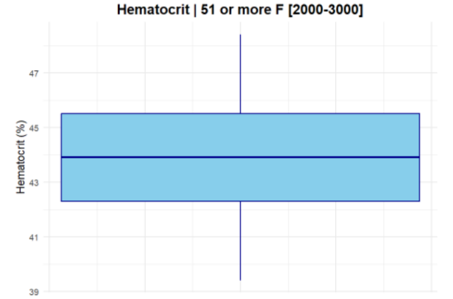

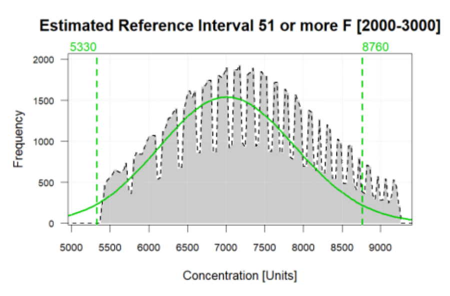

N (final): 56,505

Reference Intervals
lower limit [2.5% perc]: 5,330
upper limit [97.5% perc]: 8,760
RI [%]: 39.17 - 47.42

Subset: 18 – 64 years (M) | Altitude: [0-1100) m.a.s.l			
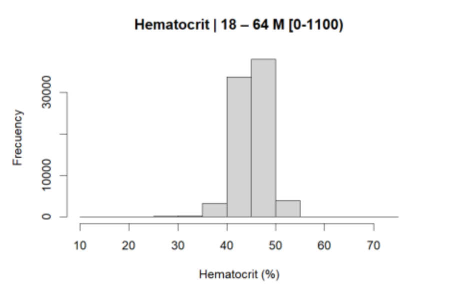	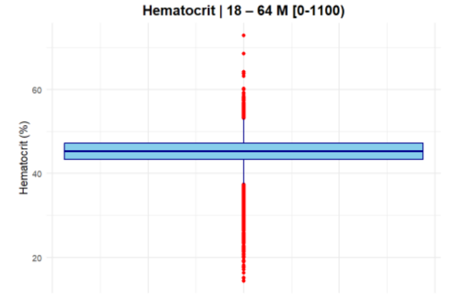	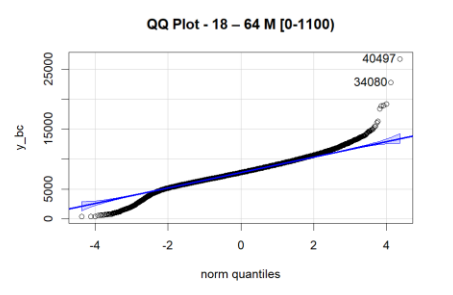	N (original): 79,547<br>Lambda Box-Cox: 2.6<br>Kolmogorov-Smirnov p: 0<br>Lilliefors p: 0<br>Normal Distribution: No<br>Outliers (Hubert): 8,944
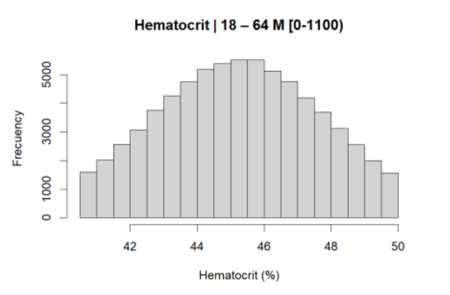	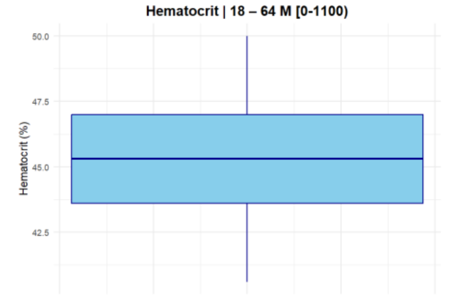	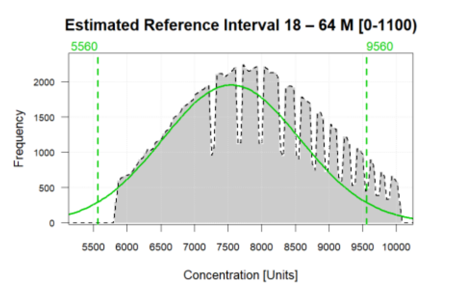	N (final): 70,603<br><br>Reference Intervals<br>lower limit [2.5% perc]: 5,560<br>upper limit [97.5% perc]: 9,560<br>RI [%]: 39.82 - 49.04
**Subset: 18 – 64 years (M) | Altitude: [1100-2000) m.a.s.l**			
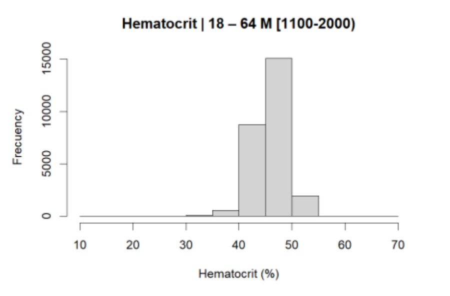	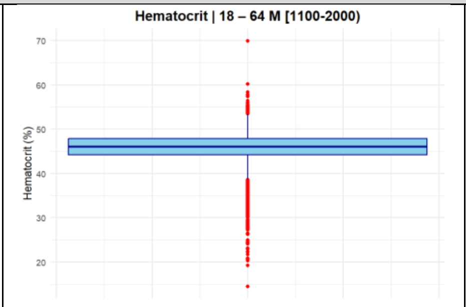	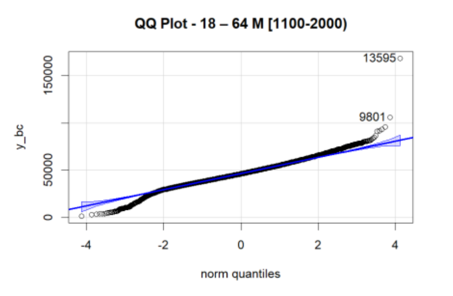	N (original): 26,451<br>Lambda Box-Cox: 3.1<br>Kolmogorov-Smirnov p: 0<br>Lilliefors p: 0<br>Normal Distribution: No<br>Outliers (Hubert): 3,131
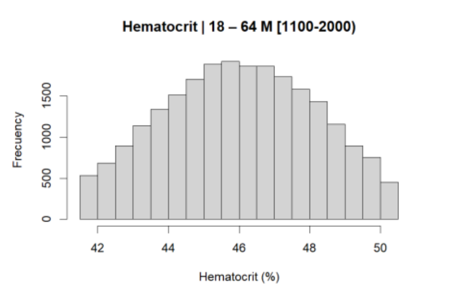	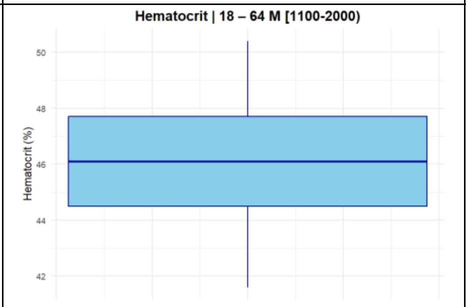	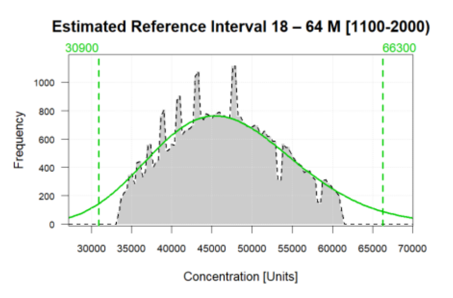	N (final): 23,320<br><br>Reference Intervals<br>lower limit [2.5% perc]: 30,900<br>upper limit [97.5% perc]: 66,300<br>RI [%]: 40.45 - 51.74

Subset: 18 – 64 years (M) | Altitude: [2000-3000] m.a.s.l

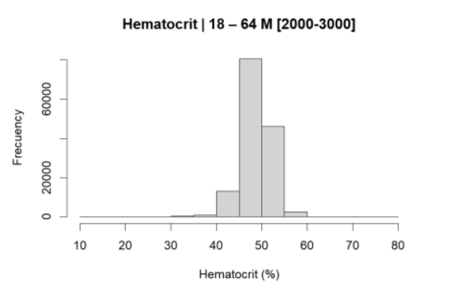

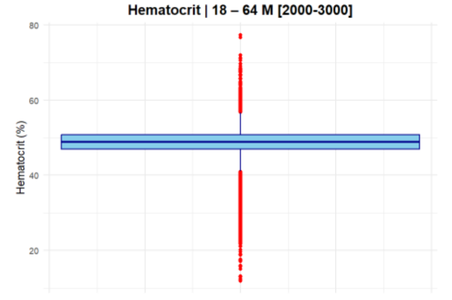

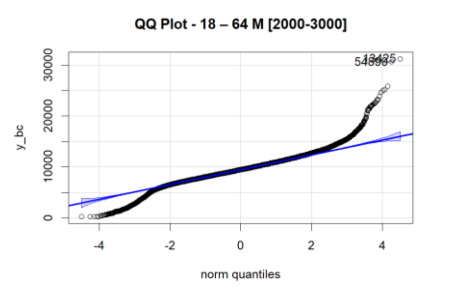

N (original): 143,797
Lambda Box-Cox: 2.6
Kolmogorov-Smirnov p: 0
Lilliefors p: 0
Normal Distribution: No
Outliers (Hubert): 18,918

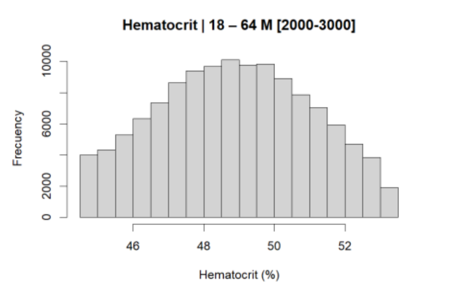

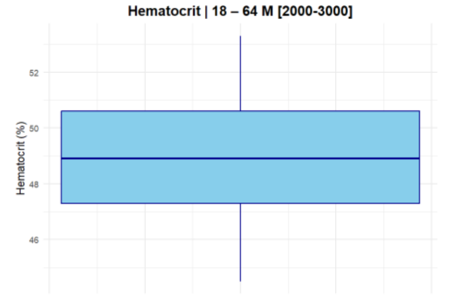

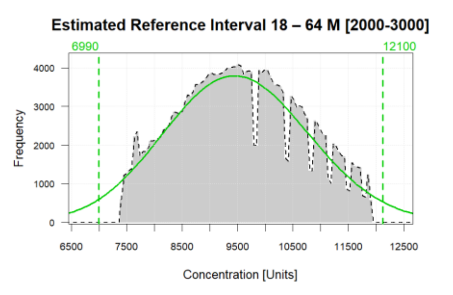

N (final): 124,879

Reference Intervals
lower limit [2.5% perc]: 6,990
upper limit [97.5% perc]: 12,100
RI [%]: 43.48 - 53.73

Subset: >64 years (M) | Altitude: [0-1100) m.a.s.l

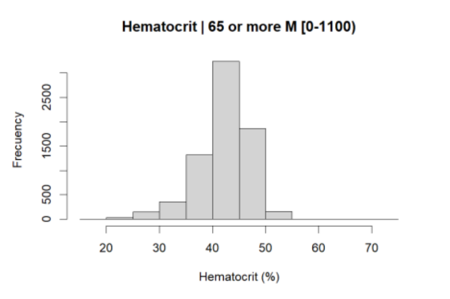

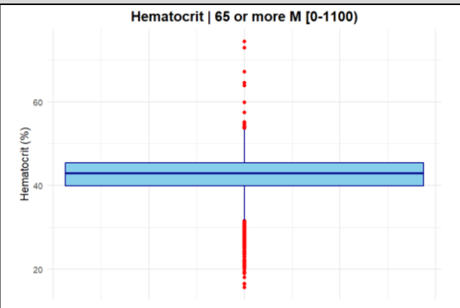

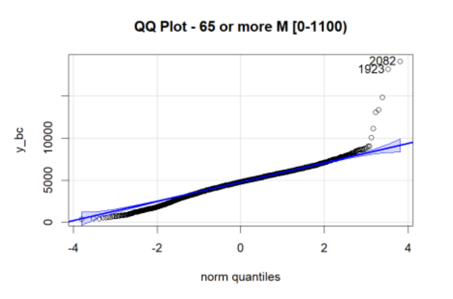

N (original): 7,143
Lambda Box-Cox: 2.5
Kolmogorov-Smirnov p: 0
Lilliefors p: 0
Normal Distribution: No
Outliers (Hubert): 968

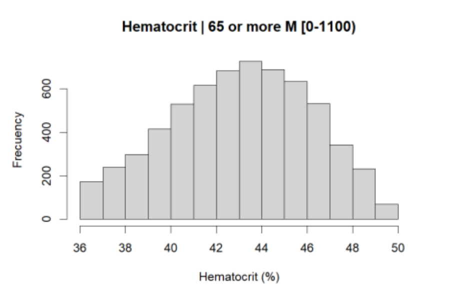

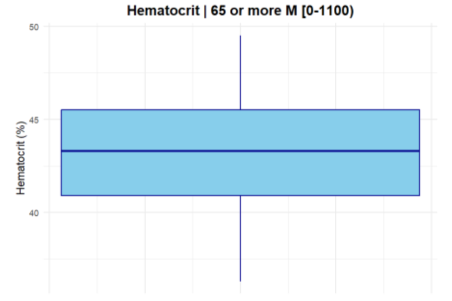

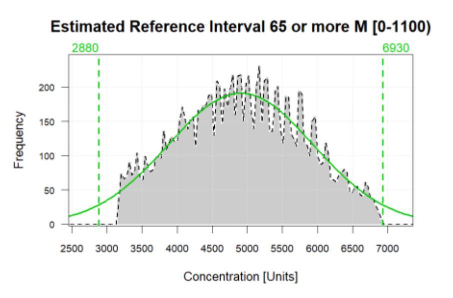

N (final): 6,175

Reference Intervals
lower limit [2.5% perc]: 2,880
upper limit [97.5% perc]: 6,930
RI [%]: 34.92 - 49.61

Subset: >64 years (M) | Altitude: [1100-2000) m.a.s.l

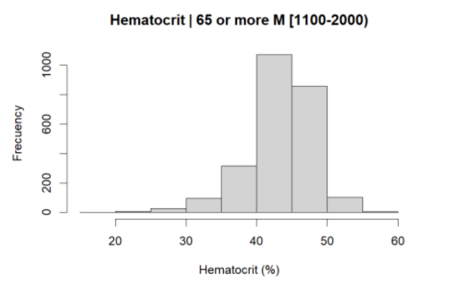

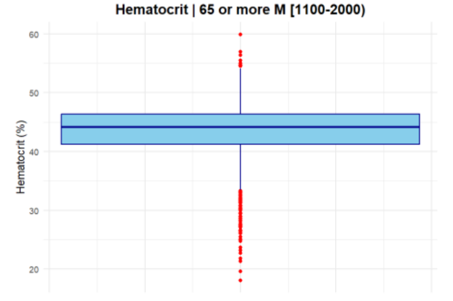

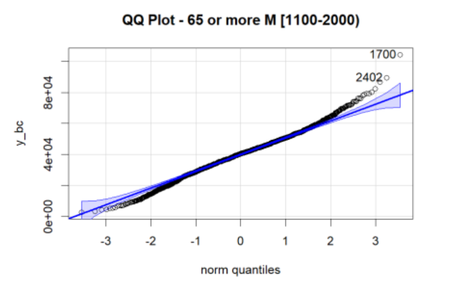

N (original): 2,479
Lambda Box-Cox: 3.1
Kolmogorov-Smirnov p: 0.0223
Lilliefors p: 0
Normal Distribution: No
Outliers (Hubert): 351

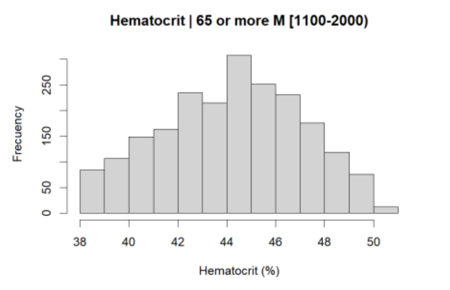

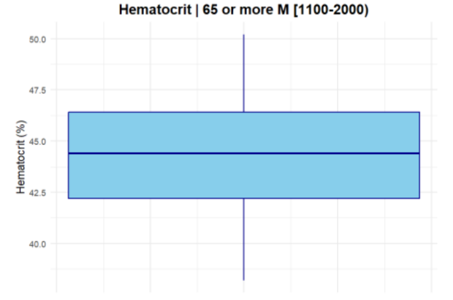

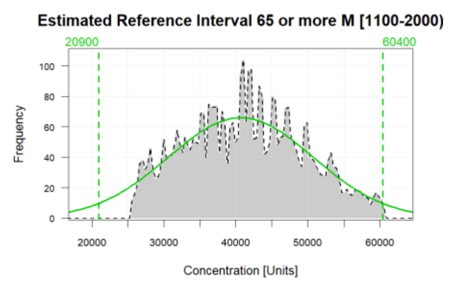

N (final): 2,128

Reference Intervals
lower limit [2.5% perc]: 20,900
upper limit [97.5% perc]: 60,400
RI [%]: 35.67 - 50.22

Subset: >64 years (M) | Altitude: [2000-3000] m.a.s.l

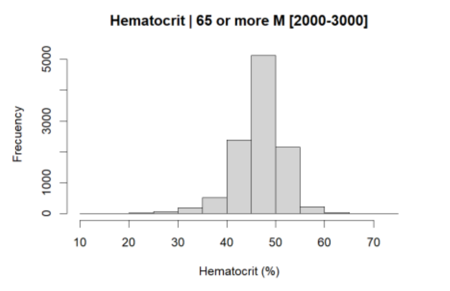

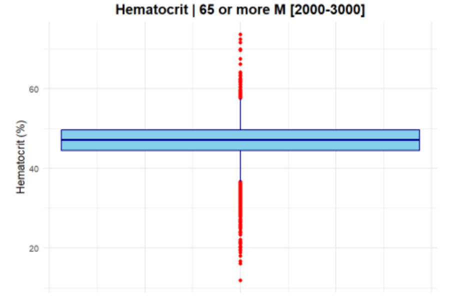

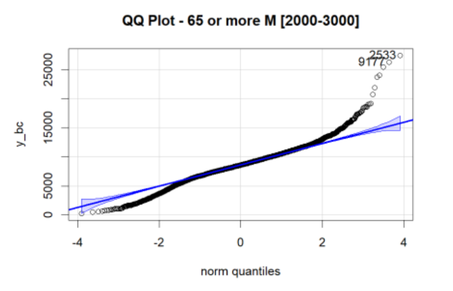

N (original): 10,764
Lambda Box-Cox: 2.6
Kolmogorov-Smirnov p: 0
Lilliefors p: 0
Normal Distribution: No
Outliers (Hubert): 1,547

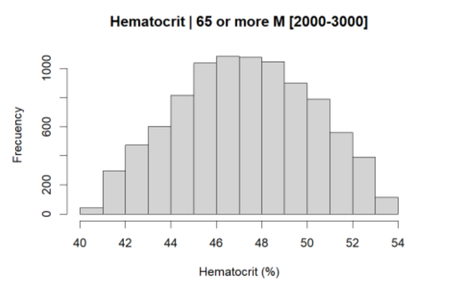

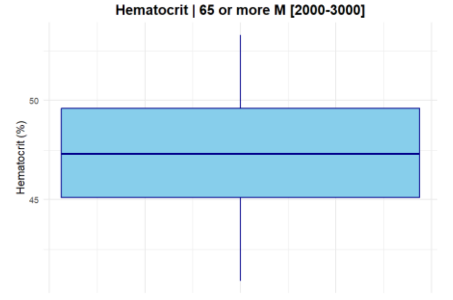

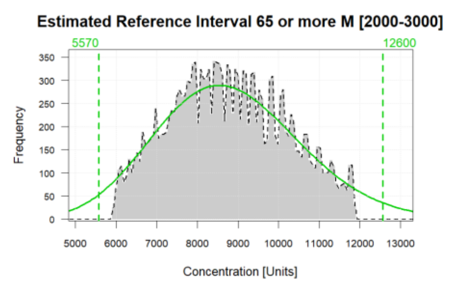

N (final): 9,217

Reference Intervals
lower limit [2.5% perc]: 5,570
upper limit [97.5% perc]: 12,600
RI [%]: 39.84 - 54.49
