# Supplementary material for: Reference Intervals for Hemoglobin and Hematocrit Adjusted for Altitude, Sex, and Age: A Big Data-Based Study in the Colombian Population
Source: Med Sci (Basel). 2026 Mar 14;14(1):136. doi: 10.3390/medsci14010136 (PMC13027793; doi:10.3390/medsci14010136)
Supplement: Supplementary file 1 [file medsci-14-00136-s001.zip › S3. ANOVA Results Hb.pdf]

S3 Table. ANOVA results for hemoglobin (Hb) by age group and altitude.

Subset: 18 – 50 years (F) | Altitude: [0-1100) m.a.s.l

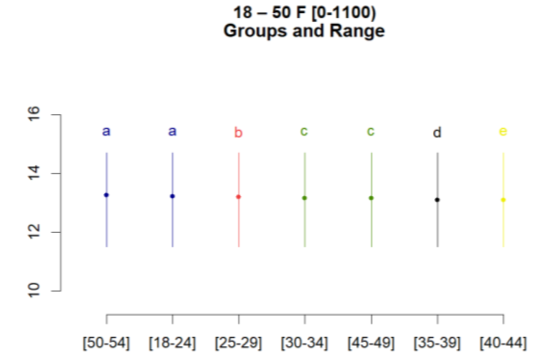

p-value ANOVA: 0

Mean groups		
[50-54]	13.25664	a
[18-24]	13.22174	a
[25-29]	13.19472	b
[30-34]	13.15634	c
[45-49]	13.14627	c
[35-39]	13.09911	d
[40-44]	13.08128	e

Group	N	RI [g/dL]
18 – 50 F [0-1100) [50-54]	1861	11,82-14,60
18 – 50 F [0-1100) [25-29]	19173	11,49-14,51
18 – 50 F [0-1100) [30-34]	19369	11,40-14,69
18 – 50 F [0-1100) [35-39]	16479	11,14-14,56
18 – 50 F [0-1100) [40-44]	14948	11,38-15,08

Subset: 18 – 50 years (F) | Altitude: [1100-2000) m.a.s.l

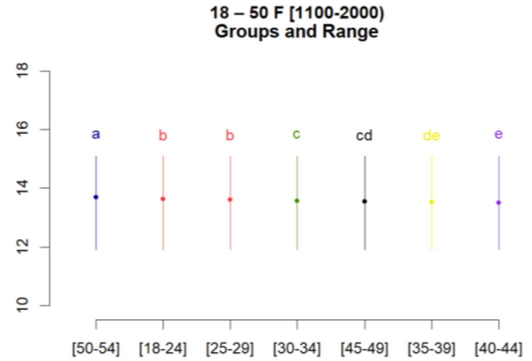

p-value ANOVA: 0

Mean groups		
[50-54]	13.69680	a
[18-24]	13.62720	b
[25-29]	13.60265	b
[30-34]	13.57650	c
[45-49]	13.54974	cd
[35-39]	13.53311	de
[40-44]	13.51054	e

Group	N	RI [g/dL]
18 – 50 F [1100-2000) [50-54]	719	12,20-14,99
18 – 50 F [1100-2000) [18-24]	7486	12,35-15,61
18 – 50 F [1100-2000) [30-34]	6754	12,08-15,44
18 – 50 F [1100-2000) [45-49]	3916	12,19-14,94
18 – 50 F [1100-2000) [35-39]	5929	12,09-14,34
18 – 50 F [1100-2000) [40-44]	5353	11,88-14,37

Subset: 18 – 50 years (F) | Altitude: [2000-3000] m.a.s.l

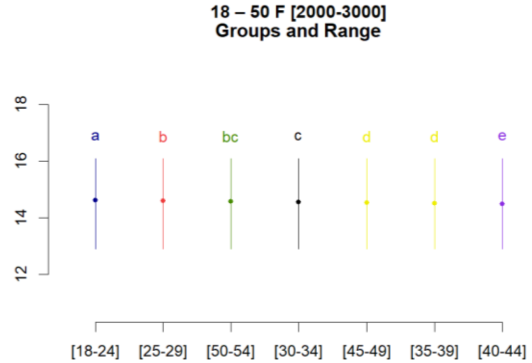

p-value ANOVA: 0

Mean groups		
[18-24]	14.63235	a
[25-29]	14.59457	b
[50-54]	14.58588	bc
[30-34]	14.56339	c
[45-49]	14.52455	d
[35-39]	14.52192	d
[40-44]	14.49286	e

Group	N	RI [g/dL]
18 – 50 F [2000-3000] [18-24]	39026	13,53-15,34
18 – 50 F [2000-3000] [25-29]	37923	13,44-15,40
18 – 50 F [2000-3000] [50-54]	3577	12,74-15,72
18 – 50 F [2000-3000] [30-34]	35757	13,13-15,59
18 – 50 F [2000-3000] [45-49]	20133	12,57-15,94
18 – 50 F [2000-3000] [40-44]	26855	12,83-15,58

Subset: 18 – 64 years (M) | Altitude: [0-1100) m.a.s.l

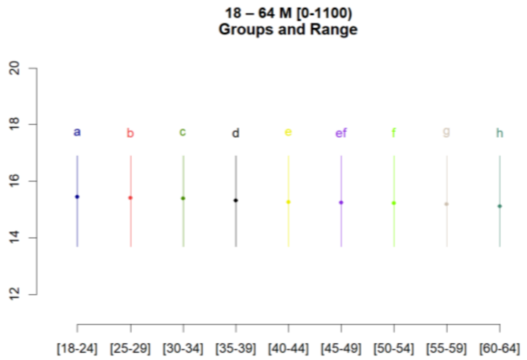

p-value ANOVA: 0

Mean groups

[18-24]	15.44282	a
[25-29]	15.41616	b
[30-34]	15.38464	c
[35-39]	15.31346	d
[40-44]	15.26186	e
[45-49]	15.23909	ef
[50-54]	15.21897	f
[55-59]	15.18492	g
[60-64]	15.11893	h

Group	N	RI [g/dL]
18 – 64 M [0-1100) [18-24]	11188	13,66-17,40
18 – 64 M [0-1100) [25-29]	9461	13,83-17,23
18 – 64 M [0-1100) [30-34]	10552	13,68-17,34
18 – 64 M [0-1100) [35-39]	10072	13,50-17,21
18 – 64 M [0-1100) [40-44]	9520	13,46-17,17
18 – 64 M [0-1100) [45-49]	6751	13,50-17,18
18 – 64 M [0-1100) [50-54]	5445	14,50-15,64
18 – 64 M [0-1100) [55-59]	4768	14,63-15,81
18 – 64 M [0-1100) [60-64]	3867	13,77-16,52

Subset: 18 – 64 years (M) | Altitude: [1100-2000) m.a.s.l

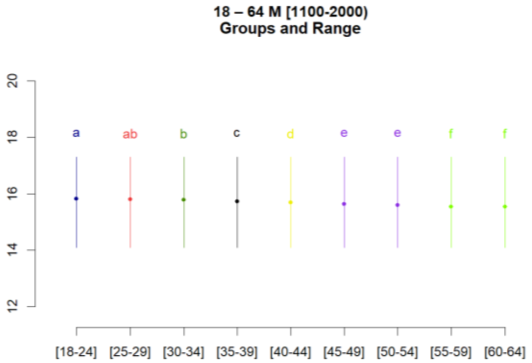

p-value ANOVA: 0

Mean groups

[18-24]	15.82281	a
[25-29]	15.80842	ab
[30-34]	15.77710	b
[35-39]	15.73408	c
[40-44]	15.68948	d
[45-49]	15.64242	e
[50-54]	15.60481	e
[55-59]	15.54716	f
[60-64]	15.54424	f

Group	N	RI [g/dL]
18 – 64 M [1100-2000) [18-24]	3901	14,49-17,31
18 – 64 M [1100-2000) [25-29]	3361	14,33-16,99
18 – 64 M [1100-2000) [30-34]	3468	13,98-17,11
18 – 64 M [1100-2000) [35-39]	3137	13,97-16,98
18 – 64 M [1100-2000) [40-44]	2938	14,19-17,12
18 – 64 M [1100-2000) [45-49]	2176	14,01-17,46
18 – 64 M [1100-2000) [55-59]	1639	13,72-17,46

Subset: 18 – 64 years (M) | Altitude: [2000-3000] m.a.s.l

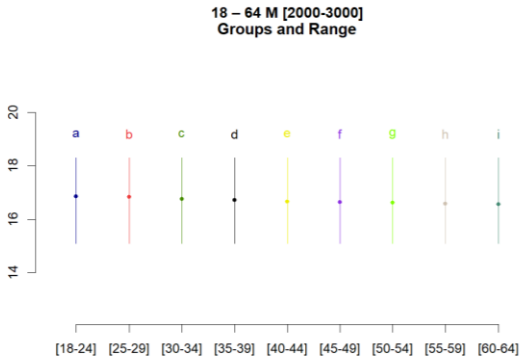

p-value ANOVA: 0

Mean groups

[18-24]	16.85120	a
[25-29]	16.82514	b
[30-34]	16.76311	c
[35-39]	16.71023	d
[40-44]	16.66439	e
[45-49]	16.64431	f
[50-54]	16.61650	g
[55-59]	16.58126	h
[60-64]	16.55562	i

Group	N	RI [g/dL]
18 – 64 M [2000-3000] [18-24]	19956	15,13-18,62
18 – 64 M [2000-3000] [25-29]	18155	15,17-18,93
18 – 64 M [2000-3000] [30-34]	18084	15,10-18,49
18 – 64 M [2000-3000] [35-39]	16595	14,89-18,58
18 – 64 M [2000-3000] [40-44]	16046	14,80-18,59
18 – 64 M [2000-3000] [45-49]	12142	14,82-18,68
18 – 64 M [2000-3000] [50-54]	10263	14,72-18,63
18 – 64 M [2000-3000] [55-59]	9052	15,63-16,79
18 – 64 M [2000-3000] [60-64]	7077	15,28-16,66
