# Supplementary material for: Reference Intervals for Hemoglobin and Hematocrit Adjusted for Altitude, Sex, and Age: A Big Data-Based Study in the Colombian Population
Source: Med Sci (Basel). 2026 Mar 14;14(1):136. doi: 10.3390/medsci14010136 (PMC13027793; doi:10.3390/medsci14010136)
Supplement: Supplementary file 1 [file medsci-14-00136-s001.zip › S4. ANOVA Results Hct.pdf]

S4 Table. ANOVA results for hematocrit (Hct) by age group and altitude.

Subset: 18 – 50 years (F) | Altitude: [0-1100) m.a.s.l

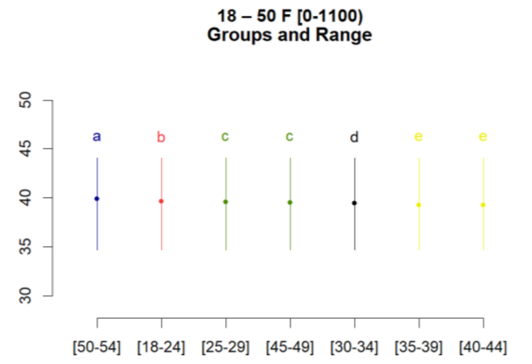

p-value ANOVA: 0

	Mean	groups
[50-54]	39.87367	a
[18-24]	39.67123	b
[25-29]	39.55401	c
[45-49]	39.51189	c
[30-34]	39.45091	d
[35-39]	39.26591	e
[40-44]	39.23229	e

Group	N	RI [%]
18 – 50 F [0-1100) [50-54]	1865	34,42-45,17
18 – 50 F [0-1100) [18-24]	19829	36,58-44,66
18 – 50 F [0-1100) [25-29]	18815	35,52-44,98
18 – 50 F [0-1100) [30-34]	19053	34,35-44,80
18 – 50 F [0-1100) [35-39]	16475	35,33-44,59

Subset: 18 – 50 years (F) | Altitude: [1100-2000) m.a.s.l

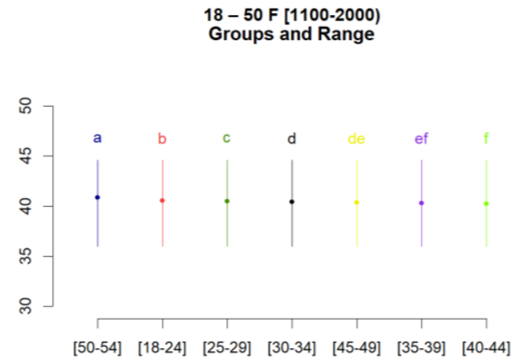

p-value ANOVA: 0

	Mean	groups
[50-54]	40.86503	a
[18-24]	40.55886	b
[25-29]	40.45412	c
[30-34]	40.38170	d
[45-49]	40.37890	de
[35-39]	40.30177	ef
[40-44]	40.24274	f

Group	N	RI [%]
18 – 50 F [1100-2000) [50-54]	692	35,48-46,58
18 – 50 F [1100-2000) [18-24]	7256	34,96-45,59
18 – 50 F [1100-2000) [25-29]	6761	35,14-45,25
18 – 50 F [1100-2000) [30-34]	6595	35,17-44,82
18 – 50 F [1100-2000) [45-49]	3863	35,27-45,88
18 – 50 F [1100-2000) [35-39]	5829	36,20-44,81
18 – 50 F [1100-2000) [40-44]	5267	34,80-45,20

Subset: 18 – 50 years (F) | Altitude: [2000-3000] m.a.s.l

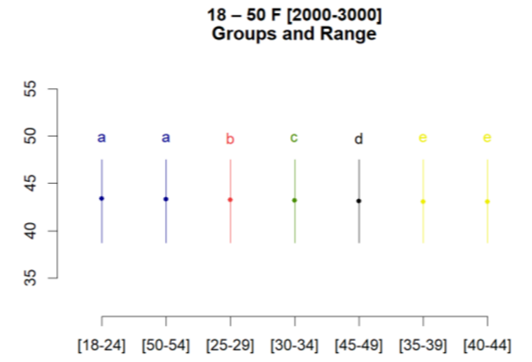

p-value ANOVA: 0

	Mean	groups
[18-24]	43.39584	a
[50-54]	43.34777	a
[25-29]	43.26299	b
[30-34]	43.18425	c
[45-49]	43.13656	d
[35-39]	43.08130	e
[40-44]	43.05058	e

Group	N	RI [%]
18 – 50 F [2000-3000] [18-24]	38433	38,39-47,59
18 – 50 F [2000-3000] [25-29]	37278	38,21-47,59
18 – 50 F [2000-3000] [30-34]	35129	40,18-47,59
18 – 50 F [2000-3000] [45-49]	20062	37,69-48,25
18 – 50 F [2000-3000] [35-39]	29842	40,08-47,52

Subset: 18 – 64 years (M) | Altitude: [0-1100) m.a.s.l

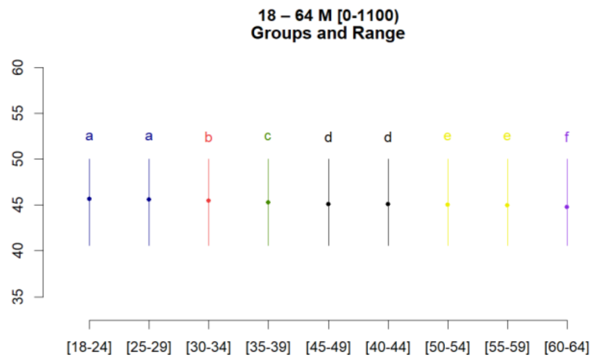

p-value ANOVA: 0

Mean groups

[18-24] 45.66287 a
[25-29] 45.60127 a
[30-34] 45.47065 b
[35-39] 45.26204 c
[45-49] 45.10303 d
[40-44] 45.09919 d
[50-54] 44.98616 e
[55-59] 44.95040 e
[60-64] 44.74347 f

Group	N	RI [%]
18 – 64 M [0-1100) [18-24]	11052	40,31-51,76
18 – 64 M [0-1100) [30-34]	10431	40,11-51,14
18 – 64 M [0-1100) [35-39]	9953	40,04-51,07
18 – 64 M [0-1100) [45-49]	6599	40,08-50,59
18 – 64 M [0-1100) [50-54]	5374	39,28-50,66
18 – 64 M [0-1100) [60-64]	3825	36,55-50,26

Subset: 18 – 64 years (M) | Altitude: [1100-2000) m.a.s.l

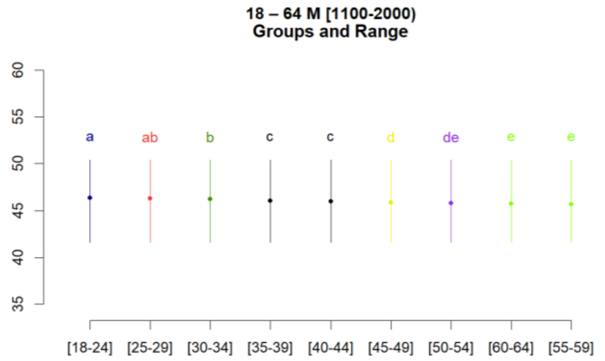

p-value ANOVA: 0

Mean groups

[18-24] 46.35813 a
[25-29] 46.28214 ab
[30-34] 46.21561 b
[35-39] 46.00718 c
[40-44] 45.98697 c
[45-49] 45.86175 d
[50-54] 45.80465 de
[60-64] 45.71014 e
[55-59] 45.68017 e

Group	N	RI [%]
18 – 64 M [1100-2000) [18-24]	3874	41,16-51,22
18 – 64 M [1100-2000) [25-29]	3281	42,17-51,46
18 – 64 M [1100-2000) [30-34]	3375	40,99-51,97
18 – 64 M [1100-2000) [35-39]	3064	41,09-51,45
18 – 64 M [1100-2000) [45-49]	2136	40,82-51,23
18 – 64 M [1100-2000) [50-54]	1765	42,10-50,40
18 – 64 M [1100-2000) [60-64]	1322	40,29-51,17

Subset: 18 – 64 years (M) | Altitude: [2000-3000] m.a.s.l

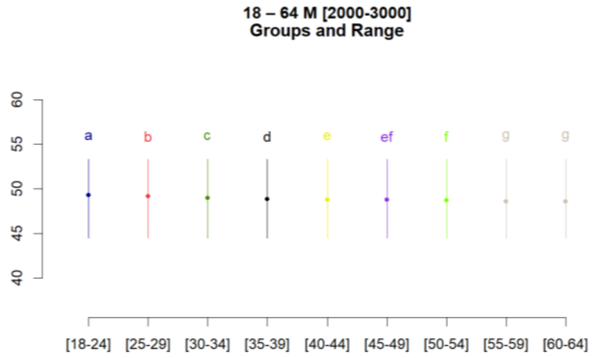

p-value ANOVA: 0

Mean groups

[18-24] 49.27730 a
[25-29] 49.15571 b
[30-34] 48.99617 c
[35-39] 48.85929 d
[40-44] 48.78482 e
[45-49] 48.74263 ef
[50-54] 48.68645 f
[55-59] 48.58560 g
[60-64] 48.55233 g

Group	N	RI [%]
18 – 64 M [2000-3000] [18-24]	19777	44,65-54,54
18 – 64 M [2000-3000] [25-29]	17865	44,69-54,54
18 – 64 M [2000-3000] [30-34]	17772	44,56-54,19
18 – 64 M [2000-3000] [35-39]	16232	43,94-52,77
18 – 64 M [2000-3000] [40-44]	15655	44,06-54,12
18 – 64 M [2000-3000] [45-49]	11868	42,20-53,63
18 – 64 M [2000-3000] [50-54]	9982	41,65-54,13
18 – 64 M [2000-3000] [55-59]	8835	42,76-52,86
